# ROS-responsive exogenous functional mitochondria can rescue neural cells post-ischemic stroke

**DOI:** 10.3389/fcell.2023.1207748

**Published:** 2023-07-03

**Authors:** Yanjiao Li, Yachao Wang, Weiqi Yang, Zhen Wu, Daiping Ma, Jianxiu Sun, Huixian Tao, Qinlian Ye, Jingnan Liu, Zhaoxia Ma, Lihua Qiu, Weiping Li, Liyan Li, Min Hu

**Affiliations:** ^1^ Institute of Neuroscience, Kunming Medical University, Kunming, China; ^2^ Yunnan Key Laboratory for Basic Research on Bone and Joint Diseases and Yunnan Stem Cell Translational Research Center, Kunming University, Kunming, China; ^3^ Department of Neurosurgery, The Institute Translational Medicine, The First Affiliated Hospital of Shenzhen University, Shenzhen Second People's Hospital, Shenzhen, China; ^4^ Department of Burn Plastic Surgery, Shenzhen Second People’s Hospital, Shenzhen, China; ^5^ Yunnan Jici Institute for Regenerative Medicine Co., Ltd., Kunming, China; ^6^ Department of Pathophysiology, Basic Medical and Public Health School, Jinan University, Guangzhou, China; ^7^ Department of Neurosurgery, Shenzhen Second People’s Hospital/The First Affiliated Hospital of Shenzhen University Health Science Center, Shenzhen, China; ^8^ Shenzhen Zhendejici Pharmaceutical Research and Development Co., Ltd., Shenzhen, China

**Keywords:** mitochondria internalization, mitochondria transplantation, ischemic stroke, exogenous functional mitochondria, ROS, neuroprotection

## Abstract

**Background:** The transfer of mitochondria from healthy mesenchymal stem cells (MSCs) to injured MSCs has been shown to have potential therapeutic benefits for neural cell post-ischemic stroke. Specifically, functional mitochondria can perform their normal functions after being internalized by stressed cells, leading to host cell survival. However, while this approach shows promise, there is still a lack of understanding regarding which neural cells can internalize functional mitochondria and the regulatory mechanisms involved. To address this gap, we investigated the ability of different neural cells to internalize exogenous functional mitochondria extracted from MSCs.

**Methods:** Functional mitochondria (F-Mito) isolated from umbilical cord derived-MSCs (UCMSCs) were labeled with lentivirus of HBLV-mito-dsred-Null-PURO vector. The ability of stressed cells to internalize F-Mito was analyzed using a mouse (C57BL/6 J) middle cerebral artery occlusion (MCAO) model and an oxygen-glucose deprivation/reoxygenation (OGD/R) cell model. The cell viability was measured by CCK-8 kit. Time-course of intracellular ROS levels in stressed cells were analyzed by DCFH-DA staining after OGD/R and F-Mito treatment. MitoSOX, Mitotracker and WGA labeling were used to assess the relationship between ROS levels and the uptake of F-Mito at the single-cell level. Pharmacological modulation of ROS was performed using acetylcysteine (ROS inhibitor).

**Results:** Our findings demonstrate that neurons and endothelial cells are more effective at internalizing mitochondria than astrocytes, both *in vitro* and *in vivo*, using an ischemia-reperfusion model. Additionally, internalized F-Mito decreases host cell reactive oxygen species (ROS) levels and rescues survival. Importantly, we found that the ROS response in stressed cells after ischemia is a crucial determinant in positively mediating the internalization of F-Mito by host cells, and inhibiting the generation of ROS chemicals in host cells may decrease the internalization of F-Mito. These results offer insight into how exogenous mitochondria rescue neural cells via ROS response in an ischemic stroke model. Overall, our study provides solid evidence for the translational application of MSC-derived mitochondria as a promising treatment for ischemic stroke.

## 1 Introduction

Ischemic stroke poses a significant threat to the elderly population worldwide, leading to physical disabilities, such as hemiplegia, aphasia, unconsciousness, and twitching, and often resulting in death (Lui and Nguyen, 2018). Neural cell death due to hypoxia, overproduction of free radicals, Ca^2+^ overload, and excitotoxicity occurs during the onset of ischemic stroke, leading to evident neurological deficits (Radak et al., 2017; Arumugam et al., 2018). Additionally, endothelial cell (EC) death occurs rapidly after the stroke onset, disrupting the blood-brain barrier (BBB) and lowering oxygen and nutrient supplies to the surrounding cellular environment (Krueger et al., 2015; Zille et al., 2019). After the ischemic stroke, astrocytes and microglia are activated, providing benefits for neurotrophin release, neuroprotection, lesion extension limitation via anti-excitotoxicity effects, and neurological recovery promotion. However, they can also trigger inflammation, aggravating the ischemic lesion and forming a glial scar obstructing axonal regeneration (Pekny et al., 2014; Fourgeaud et al., 2016; Zhao et al., 2017; Han et al., 2018; Inoue and Tsuda, 2018).

Mitochondria play a critical role in regulating cellular homeostasis by orchestrating various metabolic and stress response pathways, including ATP synthesis, calcium signaling, initiation of cell death, and synthesis of biomolecules (Nunnari and Suomalainen, 2012; Pekkurnaz and Wang, 2022). In addition, Mitochondria exhibit remarkable functional plasticity in a cell to meet local metabolic demands (Li et al., 2020). Intercellular functional mitochondria transfer has been observed among mesenchymal stem cells (MSCs) (Li et al., 2017), and recent studies have found that internalizing exogenous mitochondria can occur in neurons, microglia, and endothelial cells after ischemic stroke, promoting endogenous cell viability and activity (Huang et al., 2016; Hayakawa et al., 2018). However, to what extent target cells internalize free mitochondria post-ischemia stroke is unclear.

Recent findings suggest cell stress is a prerequisite for intercellular mitochondria transfer. For example, ECs or cardiomyocytes subjected to glucose-oxygen deprivation and reoxygenation acquired mitochondria from MSCs to improve survival through tunneling nanotubes (TNTs) (Liu et al., 2014; Han et al., 2016). Additionally, doxorubicin treatment increases mitochondrial transfer from endothelial progenitors to injured endothelial cells post-treatment (Yasuda et al., 2010), and ethidium bromide treatment enhances mitochondrial transfer from MSCs to osteosarcoma-treated cells (Cho et al., 2012). Acute lung injury caused by rotenone or TNF-treatment increases TNT formation for transferring mitochondria from stem cells to lung epithelial cells (Ahmad et al., 2014). However, the molecular signals by which events in stressed cells initiate the acquisition of exogenous functional mitochondria and how this process is regulated remains unclear.

In this study, we investigated the ability of different neural cells post-ischemic stroke to internalize functional mitochondria (F-Mito) extracted from umbilical blood-derived MSCs using a mouse middle cerebral artery occlusion (MCAO) model *in vivo* and an oxygen-glucose deprivation/reoxygenation (OGD/R) cell model *in vitro*. Our findings demonstrate that neurons and endothelial cells are more effective at internalizing mitochondria than astrocytes, both *in vitro* and *in vivo*, using an ischemia-reperfusion model. Additionally, internalized F-Mito decreases host cell reactive oxygen species (ROS) levels and rescues survival. Importantly, we found that the ROS response in stressed cells after ischemia is a crucial determinant in positively mediating the internalization of F-Mito by host cells, and inhibiting the generation of ROS chemicals in host cells may decrease the internalization of F-Mito. Our study provides solid evidence for the translational application of MSC-derived mitochondria as a promising cell-free treatment for ischemic stroke.

## 2 Materials and methods

### 2.1 Cell cultures

#### 2.1.1 hUCMSC cultures

The umbilical cord was obtained from full-term human placenta of a healthy donor. Informed consents were signed before collection. Umbilical cord collection procedures were carried out according to the guidelines approved by the Medical Ethics Committee of Medicine Department of Kunming University, and the ethical approval reference number was No. 2020001, Ethical Review 2021. hUCMSCs were isolated according to the procedures described by Kestendjieva S*,* et al. (Kestendjieva et al., 2008). Briefly, blood vessels in umbilical cord were carefully removed. Then wharton’s jelly was dissected into small fragments and treated with 0.2% collagenase for 2.5 h at 37°C and then treated with CTS TrypLE Select Enzyme (Gibco, USA) for 30 min at 37°C with agitation. Single cells were seeded in 25 cm^2^ flasks (Corning, USA) and maintained in culture medium including minimum essential medium-a (Biological Industries, Israel) and 5% human platelet (Biological Industries, Israel). When the confluence reached 70%–80%, cells were passaged. At passage 3, hUCMSCs were characterized according to the International Society for Cellular Therapy Guidelines.

#### 2.1.2 Primary astrocyte cultures

Primary astrocyte cultures were prepared from the cerebral cortex of 2-day-old newborn C57BL/6 J mice. Dissociated cortical cells were suspended in DMEM (DMEM, Life Technology) containing 25 mM glucose, 4 mM glutamine, 1 mM sodium pyruvate, and 10% fetal bovine serum and 1% Penicillin Streptomycin, and seeded on uncoated 25 cm^2^ flasks at a density of 6 × 10^5^ cells/cm^2^. Monolayers of astrocytes were obtained about 14 days after plating. Non-astrocytic cells such as microglia and neurons were detached from the flasks by shaking and removed by changing the medium. After the cells reached 70%–80% confluence, astrocytes were dissociated by trypsinization and then reseeded on uncoated T75 flasks.

#### 2.1.3 Primary neuron cultures

Primary neuron cultures were prepared from cerebral cortices of 2-day-old neonatal C57BL/6 J mice mouse. Briefly, the culture plates were coated with poly-D-lysine (Sigma, P7886). The cortices were dissected and dissociated using papain dissociation system.(Worthington Biochemical Corporation, LK003150). Cells were seeded at a density of 0.8–1 × 10^5^/cm^2^ and cultured in Dulbecco’s modified Eagle medium.(Life Technology, 11965084) containing 25 mM glucose, 4 mM glutamine, 1 mM.Sodium pyruvate, and 5% fetal bovine serum (Biological Industries, Israel). At 24 h after seeding, the medium was changed to Neurobasal™-A medium (Life Technology, 10888022) containing 1 mM glutamine and 1X B-27 (Life Technology, 17504044). Cells were cultured at 37°C in a humidified chamber of 95% air and 5% CO_2_. Cultures were used for experiments from 8 to 12 days after seeding.

#### 2.1.4 PC-12 and bEnd.3 cell cultures

PC-12 (cell NO. CL-0480) cells were purchased from Wuhan Procell Life Science and Technology Co., Ltd. bEnd.3 cells (cell NO. BNCC337672) were purchased from Beijing BeNa culture collection Life Science and Technology Co., Ltd. Cells were cultured in Dulbecco’s Modified Eagle Medium (DMEM, Life Technology, 11965084) supplemented with 10% fetal bovine serum (Biological Industries, Israel) and 1% Penicillin Streptomycin in a humidified atmosphere that contained 5% CO_2_ at 37 °C. All cell lines were negative by *mycoplasma* testing.

### 2.2 Labeling mitochondria in cell and isolation of the functional mitochondria (F-Mito) from the human umbilical cord-derived mesenchymal stem cells (UCMSCs)

Mitochondria in UCMSCs were labeled using either lentivirus-mediated HBLV-mito-dsred-Null-PURO (LV60102279, HANBIO) or Mito-tracker Green (C1048, Beyotime). To label the mitochondria with lentivirus of HBLV-mito-dsred-Null-PURO, UCMSCs at passage 5 were seeded into 6-well plates with a density of 2 × 10^5^ cells/well on Day-1. On day0 5 μL lentivirus and 1 μL polybrene were added into 1 mL UCMSC culture medium and the final concentration of polybrene was 5 μg/mL and after gently mixing, the mixture was added to UCMSCs (1 mL for each well). By the time of transfection, the confluency of USMSCs should be at 50%. After 12-h incubation with lentivirus, the media containing lentivirus were replaced by fresh UCMSC culture medium. The transfected cells were maintained till they reached 80% confluency and then were treated with 1 μg/mL puromycin for 3–4 days to select positive infected cells. The positive infected cells were passaged and expanded until passage 8 for mitochondrial isolation.

F-Mito were isolated from UCMSCs using mitochondria Isolation Kit for cultured cells (Solarbio, # SM0020-50T) according to manufacturer’s instruction and the resulting preparation was characterized for its functional properties. The isolated F-Mito were maintained on ice and protected from light and used immediately.

### 2.3 Oxygen-glucose deprivation and reoxygenation (OGD/R) and F-Mito treatment *in vitro*

The OGD/R experiment was conducted in an incubator maintained at 37°C, with an anaerobic gas mixture (95% N_2_, 5% CO_2_). Prior to OGD/R, cells were labeled with either Hoechst 33342 or 1 μg/mL WGA (W7024, invitrogen). The OGD/R was initiated using an oxygen-free, glucose-free OGD solution. After a 2.5 h incubation, the cultures were removed from the anaerobic device (Billups-Rothenberg Ltd., United States) and the OGD solution was replaced with normal medium of High-glucose DMEM with 10% FBS (HD) or normal medium containing well-distributed F-Mito, or containing 1.2 mM acetylcysteine (NAC, MedChemExpress). The F-Mito was isolated from 3.4 to 3.8 × 10^5^ UCMSCs, which corresponded to 2.5 × 10^4^/cm^2^ PC-12, bEnd.3, astrocyte cells and primary neurons. Following the OGD/R, the cells were allowed to recover for 3, 6, 9, 12, or 24 h in a conventional incubator for further experiments.

### 2.4 Cell viability assays

To perform the CCK-8 assay (C0038, Beyotime), 1 × 10^4^ cells (PC-12,bEnd.3 and astrocytes) or 2.5–3.5 × 10^4^ primary neurons were seeded into a 96-well plate and allowed to grow overnight. After treatment, the cells are incubated with the CCK-8 reagent for 60 min. During this incubation period, the CCK-8 reagent is reduced by the dehydrogenase enzymes in viable cells, resulting in the formation of a colored formazan product. The absorbance of the formazan product is then measured using a microplate reader (Infinite M200 pro, TECAN) at a wavelength of 450 nm.

### 2.5 Transmission electron microscope (TEM) method

The samples were fixed overnight at 4°C using 2.5% glutaraldehyde in 0.1 M PB (PH7.2), then washed with 0.1 M PB (PH7.2) three times for 7 min. Afterward, samples were postfixed with 1% OsO_4_ for 2 h, then washed with ddH_2_O three times for 7 min, followed by serial ethanol dehydration and acetone transition for 5 min, embedding in Epon 812 resin, polymerization at 60°C for 48 h. Serial sections of uniform thickness, 800 nm for semithin sections and 60 nm for ultrathin sections, were made using a leica EM UC7 ultramicrotome. Ultrathin sections were then loaded onto Cu grids and double stained with 2% uranyl acetate and lead citrate before observations employing a JEM-1400 Plus transmission electron microscope at 80 kv.

### 2.6 ATP measurement

Extracellular ATP is measured by CellTiter-Glo luminescence (PromegaG7570) according to the manufacture’s instruction. In simple terms, opaque-walled 96-well plates with isolated mitochondria in 50 μL fresh astrocyte culture medium (AM) or 0.2 μm filter depleted extracellular mitochondria in 50 μL AM (md-AM) were prepared. CellTiter-Glo luminescence test solution (50 μL) was added and incubated for 30 min at room temperature. Luminescent signal was determined by luminescence microplate reader.

### 2.7 Mitochondria membrane potential measurement

To monitor mitochondrial health, JC-1 dye (invitrogen) was used to assess mitochondrial membrane potential. UCMSCs or F-Mito were incubated with JC1 (5 μM or 1 μM) for 20 min at 37°C. JC1 dye exhibits potential-dependent accumulation in mitochondria, indicated by fluorescence emission shift from green (Ex 485 nm/Em 516 nm).to red (Ex 579 nm/Em 599 nm).

Mitochondria membrane potential (MMP) in UCMSCs was determined by the flow cytometry and fluorescence microscopy of SLIDEVIEW (VS200, OLYMPUS) and MMP in F-Mito was detected by fluorescence microscopy of SLIDEVIEW (VS200, OLYMPUS).

### 2.8 Mitochondrial enzymatic assays and mtDNA copy number

5 × 10^6^ UCMSCs and F-Mito from 5 × 10^6^ UCMSCs were used to measure Citrate synthase activity or mitochondria complex Ⅳ activity respectively. Citrate synthase (CS) activity was measured by citroyl synthetase kit (Nanjing Jiancheng Bioengineering Institute, Cat No. A108-1) according to the manufacturer’s instructions. And mitochondria complex Ⅳ activity was measured by Mitochondria Complex Ⅳ kit (MLBIO, Cat No. mI076308).

1 × 10^6^ UCMSCs were used to extract DNA by DNA extraction kit (TIANGEN, Cat No. YDP304-03). mtDNA copy number was determined in triplicates, on two plates in parallel, using multiplex qPCR chemistry that simultaneously amplifies a mitochondrial (ND1) and a nuclear (RNAseP) amplicon to determine their relative abundance (Picard et al., 2018). The sequences for the ND1 amplicon are as follows:

Forward primer (300 nM), 5′CCC​TAA​AAC​CCG​CCA​CAT​CT3’;

Reverse primer (300 nM): 5′GAG​CGA​TGG​TGA​GAG​CTA​AGG​T3’; and Probe (100 nM): 5′FAM-CCATCACCCTCTACATCACCGCCC-TAMRA3’.

The RNAseP assay is VIC-labeled and commercially available as a kit (Thermo, Cat No.4403328). Taqman Universal Mastermix (Takara, CatNo. RR390A) was used and the assay ran on a Quantstudio 5 real-time PCR thermocycler. mtDNA copy number was calculated as *mtDNAcn* = *[2* ^*(RNAseP Ct - ND1 Ct)*^] *× 2.*

### 2.9 Detection of ROS generation

The intracellular ROS levels were evaluated by detecting the oxidative conversion of cell-permeable (2′,7′-dichlorodihydrofluorescein diacetate dye (DCFH-DA) to fluorescent dichlorofluorescein (DCF). PC-12, bEnd.3 and astrocytes were seeded at a density of 0.5 × 10^4^–1 × 10^4^ cells per well, and primary neurons were seeded at a density of 2.5–3.5 × 10^4^ per well in 96-well culture plates. Prior to the assay, the cells were rinsed with D-Hank’s medium and then incubated with DCFH-DA at 37°C for 30 min. The DCF fluorescence intensity of the cells was measured using a microplate reader (Infinite M200 pro, TECAN) at an excitation wavelength of 488 nm and an emission wavelength of 525 nm.

For ROS detection in brain tissues by a confocal laser scanning microscope, the methods were adapted from previously published protocols for tissues, with some modifications (Zhao et al., 2019). Briefly, the mice at day 3 were euthanized and dehematized. The fresh brain was collected, and then sliced into 1 mm thick coronal sections. The brain sections were stained with DCFH-DA according to the manual instruction (Beyotime, Cat NO.S0033M). After the staining, the 1 mm sections were further sliced into 25 μm thick coronal sections using a CryoStar™ NX50 Cryostat (Thermo Fisher Scientific, Waltham, USA) and then visualized using a confocal laser scanning microscope (Zeiss).

For ROS detection in brain tissues by a microplate reader, the mice at day 3 were euthanized and dehematized. The fresh brain was divided into injured side and normal side as previously method with some modifications (Zhao et al., 2020). Both sides were weighted respectively, and then were homogenized and stained with DCFH-DA according to the manual instruction (Beyotime, Cat NO.S0033M). The DCF fluorescence intensity of brain tissue was measured using a microplate reader (Infinite M200 pro, TECAN).

Mitochondrial ROS production in cells was also assessed using MitoSOX red probe in accordance with the manufacturer’s instructions (M36008, Life Technologies). Briefly, cells were grown on gelatin-precoated cell climbing slides (YA0350, Solarbio) and subjected to the same treatment described above. At the 6 h and 9 h after treatment cells were rinsed once with PBS and incubated with 5 μM MitoSOX Red in medium for 30 min at 37°C. Following three washes with PBS at room temperature, cells were analyzed by SLIDEVIEW (VS200, OLYMPUS).

### 2.10 Fluorescence quantification

Cells with the marker of interest were identified using SLIDEVIEW (VS200, OLYMPUS). Mean fluorescence intensity of Mitotracker Green for F-Mito or MitoSOX red for intracellular ROS levels was quantified in 300 random cell samples of MitoSOX red positive. Background was first corrected by segmenting images so that the entireties of cells were broadly outlined and averaging fluorescence signal from the background and then subtracted it from the images. This procedure was repeated for all images at each channel. Next, cells that divided, balled up, or displayed some abnormality (e.g., double-nucleated, abnormally large) were excluded. A mask in the WGA blue fluorescence image was used to identify the whole cell and to calculate single cell area. Mean fluorescence intensity (mean gray value) was calculated by dividing the Mitotracker or MitoSOX integrated fluorescence density by the single cell area. We performed all segmentation and fluorescence quantitation steps using the ImageJ of 6 version by the method described previously (Anguissola et al., 2011).

### 2.11 Cell counts and cell proportion analyses

The analyses were conducted in accordance with previously described methods (Kriks et al., 2011). Specifically, percentages of F-Mito positive cells or MitoSOX positive cells in PC-12, bEnd.3, and astrocytes after OGD/R were determined at the scheduled time-point of treatment in samples obtained from three independent experiments. For quantification, images were randomly picked, and each image was assessed for the number of DAPI or Hoechst 33342 positive nuclei or WGA positive cells, followed by the enumeration of cells expressing the positive biomarkers of interest. All data are expressed as mean ± standard deviation (s.d.). To quantify F-Mito (DsRed labeled) within grafts, every the eighth section where a graft was identifiable was examined. Data are presented as estimated proportion of cells with positive markers of interest ±s.d.

### 2.12 Quantitative real-time-pcr (qRT-PCR)

Total RNA was extracted with TRIzol reagent (Takara Bio) according to manufacturer’s instructions. The RNA (1.0 μg) was reverse-transcribed to cDNA using Primescript RT reagent Kit (Takara Bio). For qRT-PCR, the cDNA was used as a template along with specific primers and SYBR Green using SYBR Premix EX TaqTMⅡ (Takara Bio). Cycling conditions were according to manufacturer’s instructions. The relative expression levels were normalized to that of the internal control (ACTIN). Primers used are listed as follows.

Mus-CLTA-F 5′-3′ ATG​CTG​TTG​ACG​GAG​TGA​TGA,

Mus-CLTA-R 5′-3′CCA​CTT​ACG​GAT​ACT​TTC​AGG​CT,

Mus-SRC-F 5′-3′CAA​TGC​CAA​GGG​CCT​AAA​TGT,

Mus-SRC-R 5′-3′TGT​TTG​GAG​TAG​TAA​GCC​ACG​A,

Mus-CD38-F 5′-3′CTC​ACT​CCT​GGT​GTG​GAC​TG,

Mus-CD38-R 5′-3′CCT​GGC​AGT​TCT​GAT​CTC​TCA​T,

Mus-actin-F 5′-3′GTG​ACG​TTG​ACA​TCC​GTA​AAG​A,

Mus-actin-R 5′-3′GCC​GGA​CTC​ATC​GTA​CTC​C.

### 2.13 Focal ischemic stroke

A transient middle cerebral artery occlusion (MCAO) was induced in male mice as previously described (Wang Y. C. et al, 2020), and our methods included randomization, blinding and statistical criteria. In brief, the male C57BL/6 J mice (8–10 weeks) were subjected to 5% isoflurane, followed by 1%–1.5% isoflurane during the whole procedure. During the operation, the rectal temperature was maintained at 37.0°C ± 0.2°C by a heating pad (ALC-HTP homeothermic system, Alcott Biotech, Shanghai, China), and regional cerebral blood flow (rCBF) of the MCA territory was monitored by laser Doppler flowmetry (Moor Instruments, Devon, UK). Focal brain ischemia was induced by inserting a monofilament (RWD, Shenzhen, China) into the right internal carotid artery and via the common carotid artery. After 30 min of occlusion, the filament was removed and the skin incision was sutured. Mice were returned to their cages. All animals had free access to food and water following the procedure. Mice with intracranial hemorrhage and those who did not show a reduction in rCBF >80% during MCAO and a recovery of rCBF >70% after 10 min of reperfusion were excluded. The animals were divided to sham (incision without ischemia induction), MCAO (incision followed by 30 min of occlusion and then reperfusion), MCAO - F-Mito groups. 6 animals in each group which were euthanized at 28 days after MCAO for behavioral battery test, MRI imaging and immunofluorescent staining, and 7–9 animals in each group which were euthanized at 3 days after MCAO for ROS detection.

### 2.14 Mitochondrial administration for stroke model *in vivo*

Functional mitochondria were extracted from 5 × 10^6^ UCMSCs per mouse and subsequently administered into the striatum (AP+1.0 mm, ML+2.0 mm, DV-2.0/2.5 mm from Bregma) using a 10 μL syringe (Hamilton, USA) and a stereotaxic device (RWD, 71000-M,China). The injection consisted of either 4 μL of F-Mito suspension or 4 μL of saline preparation. Throughout the procedure and until full recovery, the animals’ physiological parameters were closely monitored, including body temperature, heart rate, and respiratory rate.

### 2.15 fMRI

The imaging experiments of the three groups were performed on a 9.4 T (Shanghai United Imaging Healthcare Co., Ltd., China) small animal system. The animals were anesthetised with 1.5%–2% isofluran and positioned into the magnet with a laser-controlled system. The protocol consisted of a T2-weighted sequence, a diffusion weighted multi-shot EPI sequence to obtain the apparent diffusion coefficient (ADC)-maps and a 3D flow-compensated gradient echo TOF angiography (Langhauser et al., 2012). Sequence parameters were set as follows. RARE: TR = 2.5 s; effective TE = 60 ms; echo train length = 4; 4 averages; matrix size = 384 × 384; FOV = 17 × 17 mm^2^; slice thickness = 0.4 mm; measurement time (12 slices) = 6 min 40 s. EPI-Diffusion: 4 phase encoding steps, TR = 3 s; TE = 20 ms; 4 averages; matrix size = 128 × 128; slice thickness = 0.4 mm; 3 orthogonal diffusion directions with three b-values b = 0, 100 s/mm^2^, 1,000 s/mm^2^; measurement time (12 slices) = 5 min 36 s 3D-TOF: TR = 22 ms; TE = 3.9 ms; Flip angle = 40°; matrix size: 256 × 256 × 128; FOV = 16 × 16 × 16 mm^3^; measurement time = 15 min 46 s^4^.

### 2.16 Neurological scores and rota rod test

For neurological scores, a 48-point scoring system was used to evaluate neurological deficits, as detailed previously (Taninishi et al., 2016; Wang Y. C. et al, 2020). Briefly, this comprehensive scoring system assesses general status, motor deficits, and sensory deficits. The final score for each animal is the sum of the scores. with 0 = no deficit and 48 = maximal deficit.

For Rota rod test, mice were placed on an accelerating rotating rod (from 4 to 40 rpm; ENV577 M, Med Associates Inc., St Al bans, VT, USA), and the latency to fall from the rod was recorded (Liu et al., 2016). All mice were trained for 3 days before surgery.

### 2.17 Tissue collection

Mice were euthanized using Carbon Dioxide (CO_2_). Mice that need to be sacrificed were put into the euthanasia chamber and the CO_2_ flow rate displaced 30% of the chamber volume per minute. Gas flow was maintained for at least 1 min after apparent clinical death. Then the brain tissue were collected. For the ROS detection, the fresh brain tissues were placed in PBS for further DCFH-DA staining. For immunofluorescent staining, the brain tissues were fixed in 4% PFA until further processing.

### 2.18 Immunofluorescent staining

The brains of the mice were removed and fixed in PFA at 4°C overnight. Subsequently, they were transferred to a sucrose solution of gradually increasing concentration for 48–72 h. The brain samples were then sliced into 20 μm thick coronal sections using a CryoStar™ NX50 Cryostat (Thermo Fisher Scientific, Waltham, USA). For immunochemistry, the brain sections were incubated overnight with primary antibodies including anti-NeuN, anti-GFAP, anti-IBA1, anti-CD31 and anti-TuJ1 (Abcam, ab104224, ab7260, ab15690 and ab28364, and R&D, MAB1195) at 4°C overnight. The sections were rinsed with phosphate buffered saline (PBS) twice, followed by incubation with the corresponding secondary antibodies (Thermo Fisher Scientific, A11008, A11001) at room temperature for 2 h and then rinsed with PBS. Finally, the slices were covered with DAPI (ZLI-9557, ZSGB-BIO, China) and images were captured using a Zeiss fluorescence microscope (LSM 800, Germany).

### 2.19 Statistical analysis

The experiments were conducted three times independently (n = 3), and statistical analysis was performed using SPSS version 24. To assess statistical significance, two-group comparisons were analyzed using Student’s t-test, while multiple-group comparisons were analyzed using one-way analysis of variance (ANOVA) with Bonferroni correction. A *p*-value below 0.05 was considered statistically significant.

## 3 Results

### 3.1 The transplantation of the free functional mitochondria from UCMSCs decreased infarct volume and enhanced neurological functions in mice following an ischemic stroke

In this study, functional mitochondria were isolated from human umbilical cord mesenchymal stem cells (UCMSCs) that met the identification criteria proposed by the Mesenchymal and Tissue Stem Cell Committee of the International Society for Cellular Therapy in 2006 (Supplementary Figure S1). The mitochondrial DNA copy number per UCMSC was 831 ± 142. The mitochondria were not contaminated with pathogenic microorganisms and were specifically labeled with mitochondrial protein using a lentivirus (HBLV-mito-dsred-Null-PURO) with RFP (DsRed) signal (Supplementary Figures S2A, B). TEM images confirmed that the DsRed-labeled mitochondria had dense cristate and intact membranes, indicating their healthy state (Supplementary Figure S2C). Additionally, the mitochondria displayed robust ATP concentration compared to the mitochondria-depleted cell lysate (Supplementary Figure S2D). The Mitochondria membrane potential (Supplementary Figure S3), citrate synthase activity or mitochondria complex IV activity was measured in UCMSCs and F-Mito respectively and no significant reduction in F-Mito compared to that in UCMSCs (Supplementary Table S1), indicating the F-Mito had good activities.

**FIGURE 1:**
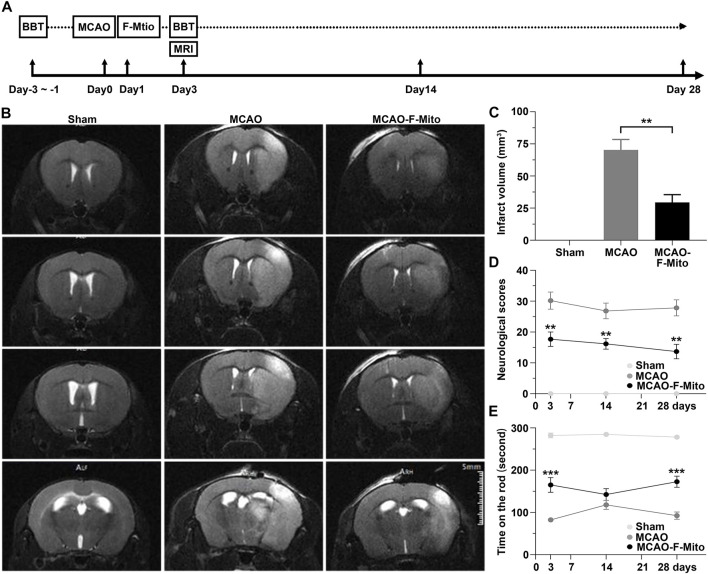
The free functional mitochondria isolated from UCMSCs transplantation decreased infarct volume and enhanced neurological functions in mice following an ischemic stroke. **(A)** The experimental design was schematically presented, where mice were subjected to transient intraluminal middle cerebral artery occlusion (MCAO) and randomly assigned to receive either saline (MCAO group) or F-Mito isolated from UCMSCs (MCAO-F-Mito group) in the lesion, starting 24 h after reperfusion. The sham group was not subjected to MCAO. The number of animals evaluated for each group (n = 6) and the time point of animal sacrifice on day 28 were provided. BBT means Behavioral Battery test including Neurological scoring and Rota rod test. **(B)** Brain MRI images of mice on day 3 post-stroke were shown. **(C)** Infarct volume analysis using MRI showed significant reduction in the MCAO-F-Mito group compared to the MCAO group. **(D)** The neurological scores of the experimental mice also demonstrated significant improvement in the MCAO-F-Mito group compared to the MCAO group, as did the results of the Rota rod experiment **(E)**. *p < 0.05, **p < 0.01, ***p < 0.001, Student’s t-test. Standard error of the mean (SEM) was reported for each group.

To assess the neuroprotection of the isolated mitochondria *in vivo*, a middle cerebral artery occlusion model (MCAO) was induced in mice (Figure 1A). The functional mitochondria were administered into the penumbra region 24 h post-stroke. Magnetic resonance imaging (MRI), neurological scores, and Rotarod tests were conducted during a 28-day observation period. The MRI analysis revealed that the infarct volume, particularly in the striatum and cortex, which appeared hyperintense in the T2-weighted images, was significantly decreased when mice were treated with F-Mito compared to the MCAO group on day 3 post-stroke (Figures 1B, C and Supplementary Figure S4). TTC staining also showed the similar results (Supplementary Figure S5). Moreover, the group treated with functional mitochondria showed a significant improvement in stroke outcomes assessed by neurological scores and Rotarod tests since 3 days post-stroke (Figures 1D, E).

### 3.2 The cellular uptake of F-Mito in the brain after an ischemic stroke

**FIGURE 2:**
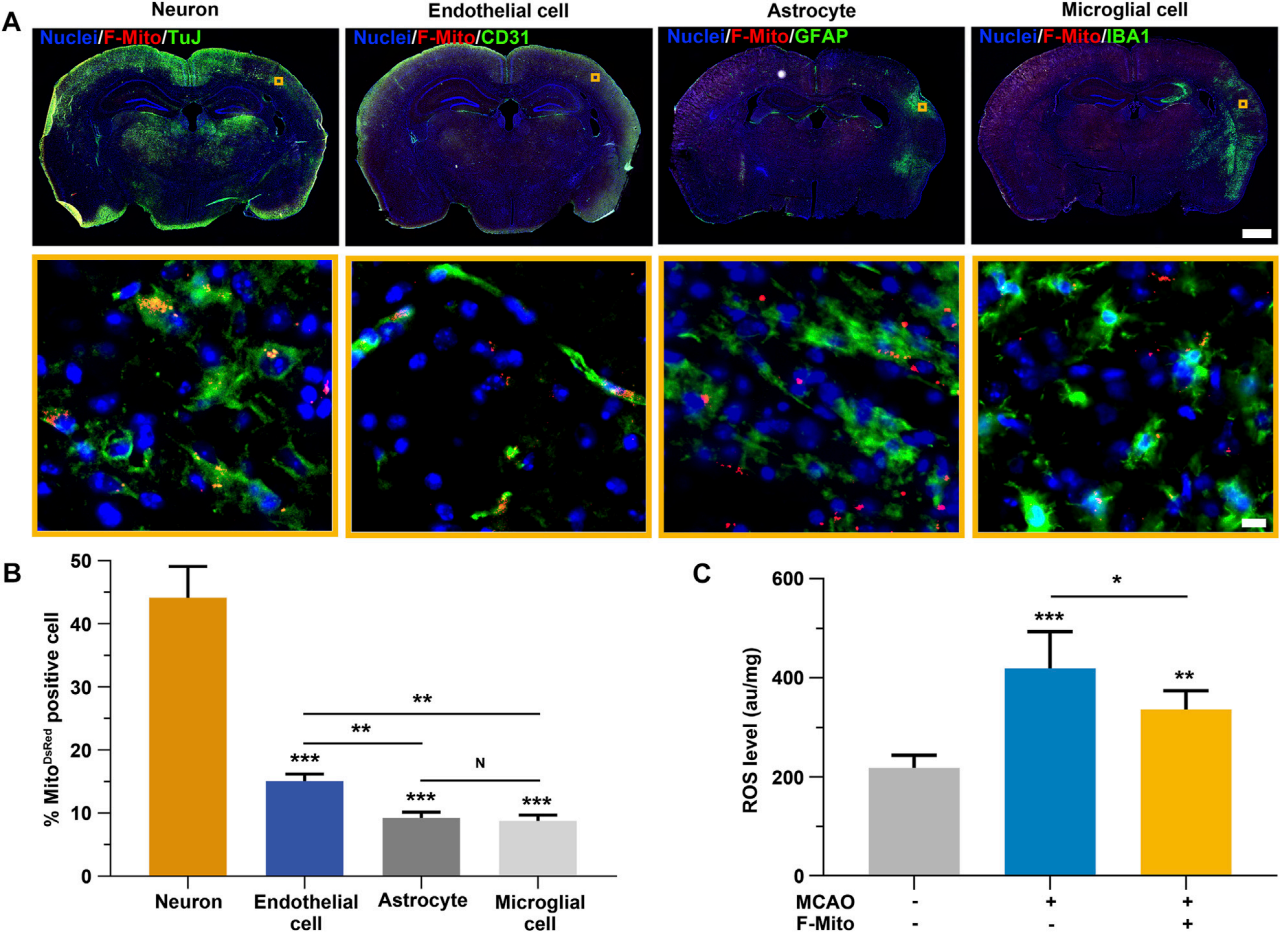
The uptake of F-Mito by neurons, endothelial cells, astrocytes and microglial cells in brain following an ischemia stroke. **(A)** The Fluorescent images demonstrated the internalization of F-Mito into cells within the lesion of the injection area. Scale bar for whole brain: 1,000 μm. Scale bar for magnification images: 10 μm. **(B)** The proportion of cells with DsRed-labeled mitochondria *in vivo* was represented, indicating statistically significant differences in comparisons made between the neuron group and other cell types, as well as between two different samples under a given line. The level of significance was denoted by *p < 0.05, **p < 0.01, and ***p < 0.001, as determined by One-way ANOVA with Bonferroni correction. The data were presented as means ± standard deviation (s.d.), and the study was conducted on six animals in the MCAO-F-Mito group. **(C)** The ROS levels of brain tissues 3 days after MCAO. The level of significance was denoted by *p < 0.05, **p < 0.01, and ***p < 0.001, as determined by One-way ANOVA with Bonferroni correction. The data were presented as means ± standard deviation (s.d.), and the study was conducted on six animals in each group.

Subsequently, we investigated the cellular uptake of F-Mito in various cell types implicated in the restoration of neurological functions following an ischemic stroke. DsRed-labeled mitochondria were distributed in neurons, endothelial cells, astrocytes, and microglia cells adjacent to the injection site using confocal microscopy (Supplementary Figure S6 and Figure 2A). Quantitative analysis revealed that F-Mito were internalized to a greater extent by neurons and endothelial cells compared to astrocytes and microglia cells, particularly in the case of neurons after a stroke (Figures 2A, B). The ROS levels were significantly decreased in F-Mito treatment group 3 days after MCAO (Figure 2C and Supplementary Figure S7). To confirm the F-Mito distribution between normal and infarcted brains, we injected F-Mito into normal brains and injured brains, and compared the percentage of the cells with F-Mito 28 days post MCAO. The results showed that the percentage was higher in the neurons and endothelial cells within damaged brains than that in the normal brains (Supplementary Figure S8).

### 3.3 The F-Mito restored cell viability and reduced intracellular ROS following OGD/R

**FIGURE 3:**
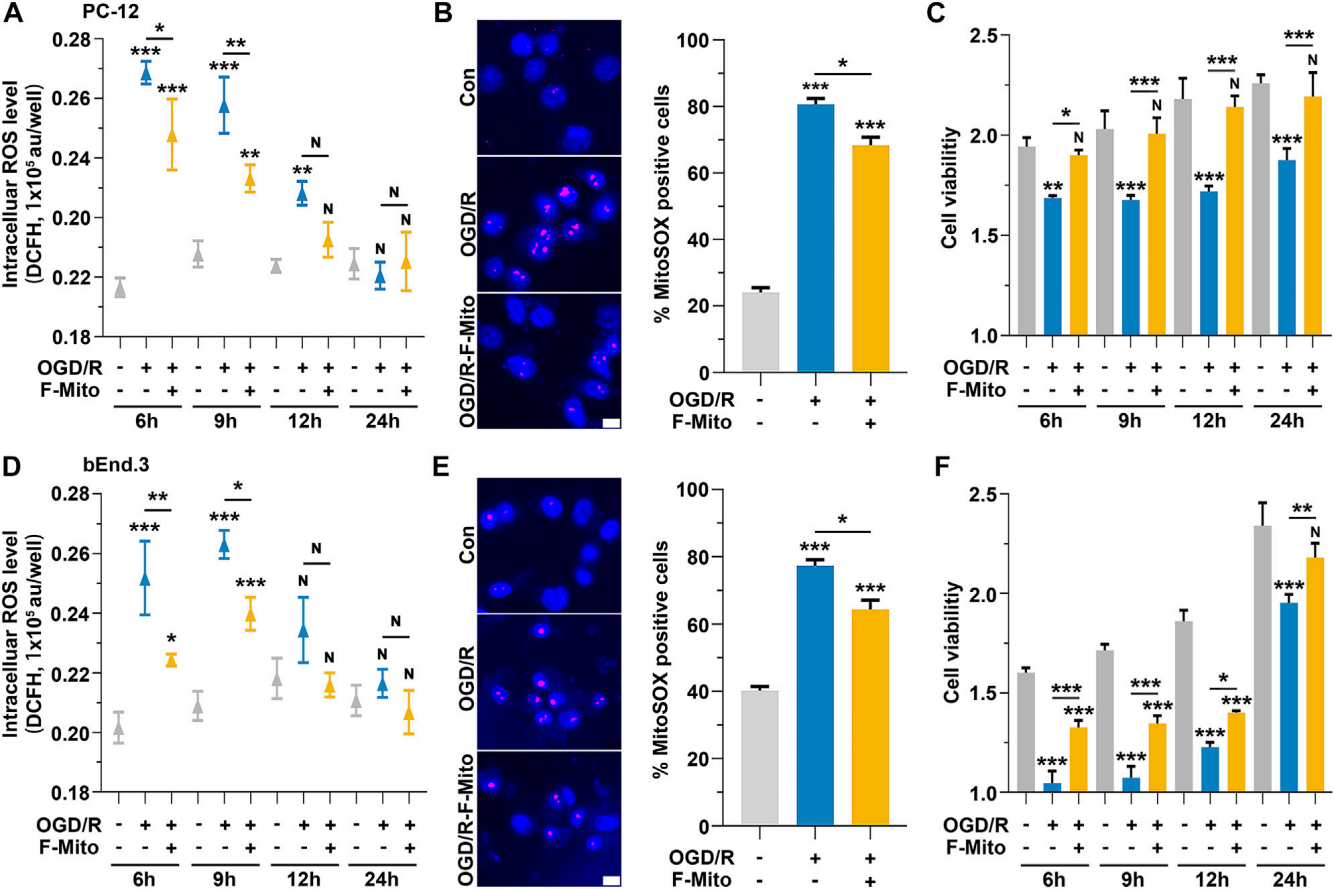
The treatment of the F-Mito reduced intracellular ROS and restored cell viability after OGD/R. The study was performed on PC-12 and bEnd.3 cells, and the results were reported for a period of 24 h after OGD/R. The treatment with F-Mito led to a restoration of cell viability and a reduction in intracellular ROS levels. **(A)** The intracellular ROS levels of PC-12 cells over time after OGD/R. **(B)** Mitochondrial ROS analysis with MitoSOX in PC-12 cells at 9 h after OGD/R. Fluorescent image of MitoSOX staining (magenta, MitoSOX; blue, WGA) and the proportion of MitoSOX-positive cells were shown. WGA was Wheat Germ Agglutinin to label cell for visualization. Scale bars: 10 μm. **(C)** The cell viability of PC-12 cells over time after OGD/R. **(D)** The intracellular ROS levels of bEnd.3 cells over time after OGD/R. **(E)** Mitochondrial ROS analysis with MitoSOX in bEnd.3 cells at 9 h after OGD/R. Scale bars: 10 μm. **(F)** The cell viability of bEnd.3 cells over time after OGD/R. In both cases, F-Mito treatment led to a significant decrease in intracellular ROS levels. Statistical analysis was conducted using One-way ANOVA with Bonferroni correction, and significance was considered when samples were compared to the control group at their respective time points (*p < 0.05, **p < 0.01, ***p < 0.001). The data were presented as means ± s.d., n = 3 independent experiments in **(A,C,D,F)**, and n > 100 cells from three independent experiments in **(B,E)**.

ROS plays a pivotal role in the pathological process during an ischemic stroke and induces cellular damage (Radermacher et al., 2013). *In vitro* ischemia-reperfusion models were employed to measure cell viability using the CCK-8 kit and ROS levels using two different dyes: DCFH-DA for peroxyl radicals and hydrogen peroxides, and MitoSOX for superoxide anions, respectively. The levels of cellular ROS were significantly increased in PC-12 cells at 6 h–12 h after exposure to oxygen-glucose deprivation and reoxygenation (OGD/R) compared to the control group cultured under normal conditions (Figure 3A). MitoSOX staining at 9 h also revealed the increase in ROS levels after cells challenged with OGD/R (Figure 3B). Correspondingly, cell viability declined over time (Figure 3C). However, following F-Mito treatment, ROS levels decreased at 6 h and 9 h compared to untreated cells and subsequently returned to normal levels at 12 h (Figures 3A, B). Likewise, cell viability was restored in F-Mito-treated cells (Figure 3C).

Similar findings were observed in bEnd.3 cells (Figures 3D–F) and in mice primary neurons (Supplementary Figure S9). In astrocytes, ROS levels began to rise at 12 h after OGD/R, and cell viability started to decrease from that point onward. However, after F-Mito intervention, ROS levels returned to normal, and cell survival was restored (Supplementary Figure S10). The above results showed that F-Mito were internalized by neurons and endothelial cells to a greater extent than astrocytes. Furthermore, F-Mito were observed to reduce intracellular ROS levels and enhance cell viability *in vitro* models of ischemia-reperfusion, indicating its potential therapeutic value in treating ischemic stroke.

### 3.4 The utilization of F-Mito increased concomitantly with the rise in ROS generation during the 24 h following OGD/R

Subsequently, we investigated whether ROS generation was accompanied by an increase in F-Mito entry. To examine this, we tracked the production of ROS and the proportion of DsRed-positive cells over a 24-h period following OGD/R.

**FIGURE 4:**
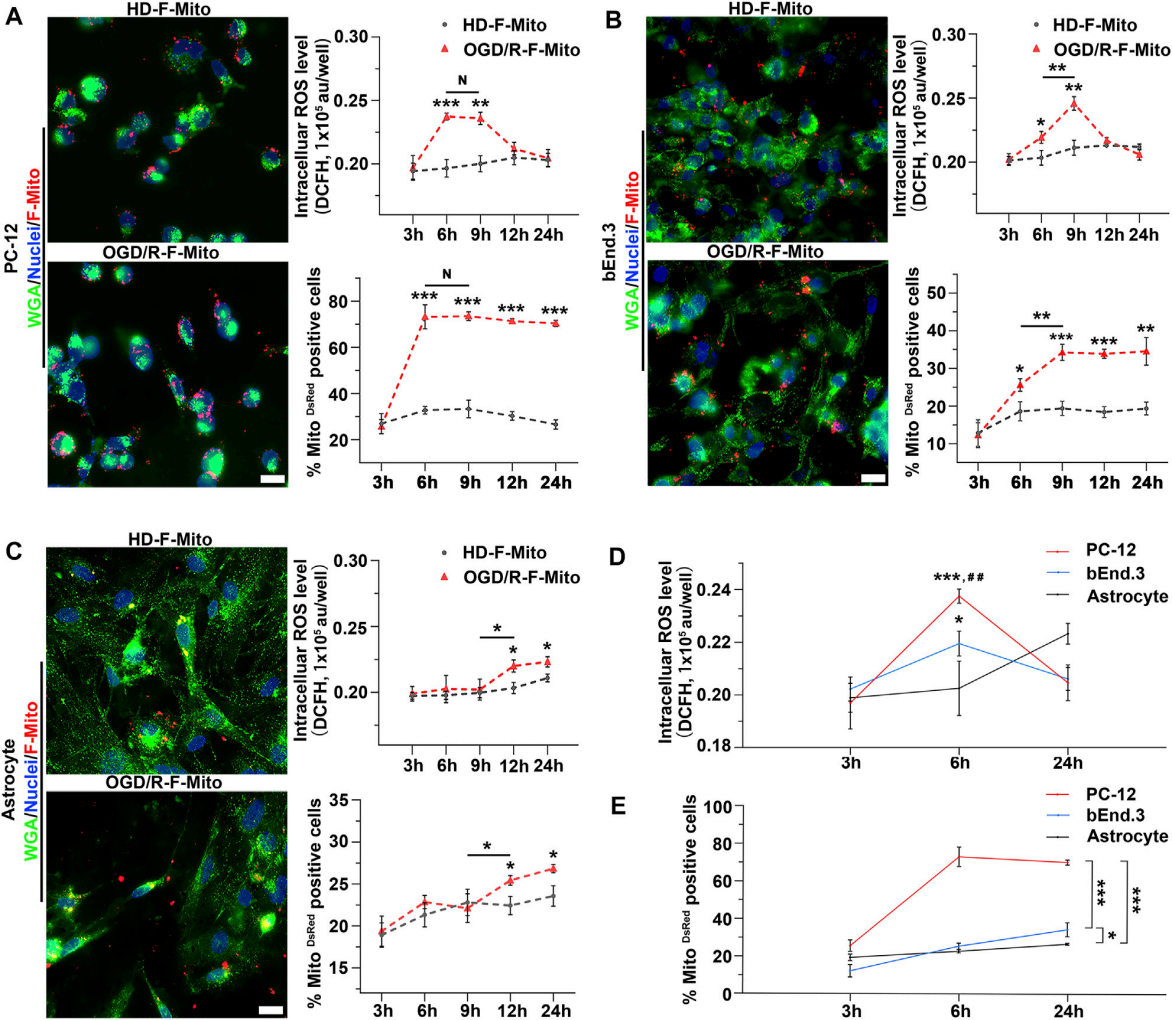
The positive dose-dependent relationship between intracellular ROS production and the uptake of F-Mito in different cell types within 24 h post OGD/R. **(A)** PC-12 cells were cultured under normal conditions and treated with F-Mito (HD-F-Mito) or subjected to OGD/R and co-cultured with F-Mito (OGD/R-F-Mito). The uptake of F-Mito by PC-12 at 6 h were shown. The intracellular ROS production within 24 h and a proportion of PC-12 cells with F-Mito over time were also depicted. WGA was used to label the cell membrane. Scale bars: 20 μm. Statistical analysis was conducted using Student’s t-test and significance was determined when sample in OGD/R-F-Mito was compared to that in HD-F-Mito group at different time point or in comparisons between two different samples under a given line (*p < 0.05, **p < 0.01, ***p < 0.001). The data were presented as means ± standard deviation (s.d.), n = 3 independent experiments in ROS detection, and n > 100 cells from three independent experiments in the analyses of the MitoDsRed positive cell ratio. **(B)** The uptake of F-Mito by bEnd.3 cells at 6 h were shown, and the intracellular ROS production within 24 h, along with the proportion of bEnd.3 cells with F-Mito over time were depicted. **(C)** The uptake of F-Mito by astrocytes at 6 h were shown, and the intracellular ROS production within 24 h, along with the proportion of astrocytes with F-Mito over time were depicted. **(D)** ROS levels in PC-12, bEnd.3, and astrocytes at 3 h, 6 h, and 24 h post OGD/R were compared. Significance was determined when samples were compared to astrocytes (*) or to bEnd.3 (#). The statistical analyses were performed using one-way ANOVA with Bonferroni correction, and the significance levels were set at *p < 0.05, **p < 0.01, and ***p < 0.001 or #*p* < 0.05, ##*p* < 0.01, ###*p* < 0.001. The data were presented as means ± s.d. and n = 3 independent experiments. **(E)** The proportion of F-Mito internalized cells in PC-12, bEnd.3, and astrocytes at 3 h, 6 h, and 24 h was compared. The data were obtained from more than 100 cells across three independent experiments.

We observed no changes in ROS levels when normal cells were treated with F-Mito, as shown in Supplementary Figure S11. In contrast, cells subjected to OGD/R and then treated with F-Mito (OGD/R-F-Mito) showed a peak in ROS levels at 6 h, accompanied by an increase in the proportion of PC-12 cells with DsRed-labeled F-Mito (Figure 4A). At 3 h post-treatment, neither ROS levels nor the proportion of cells were significantly altered compared to the normal cells treated with F-Mito (HD-Mito). However, ROS levels remained elevated at 9 h, and at the same time, the proportion of PC-12 cells with F-Mito remained constant. Although ROS levels began to decline after 9 h and returned to normal levels by 24 h, the proportion of cells with F-Mito was similar to that observed at 6 h or 9 h in PC-12 cells, indicating that internalized F-Mito were not eliminated from the stressed cells within 24 h (Figure 4A and Supplementary Figure S12A).

Similar results were obtained in primary neurons, where the peak of ROS generation occurred at 6 h (Supplementary Figure S13), and bEnd.3 cells, where the peak of ROS generation occurred at 9 h, accompanied by an increase in the uptake proportion to the maximum at the same time (Figure 4B and Supplementary Figure S12B). In contrast, ROS levels in astrocytes did not increase significantly until 12 h, and the uptake proportion also increased at 12 h (Figure 4C and Supplementary Figure S12C). Given that ROS levels were elevated as early as 6 h post-OGD/R in PC-12 cells, we further compared the proportion of F-Mito-positive cells and ROS levels in these stressed cells at 6 h and 24 h. The results showed that ROS levels were highest in PC-12 cells and lowest in astrocytes at 6 h. Correspondingly, the highest proportion of F-Mito uptake was observed in PC-12 cells, and the lowest was observed in astrocytes (Figures 4D, E). We also used flow cytometry to analyze the F-Mito distribution in different cells (Supplementary Figure S14), and the changes of F-Mito internalization ratio were consistent with the above results analyzed using fluorescent images.

These findings suggest that the higher the ROS produced in stressed cells at the early stage post-OGD/R, the higher the proportion of cells that internalize F-Mito. Furthermore, they are dose-dependent and concomitant with each other during the 24 h following OGD/R.

### 3.5 The uptake of F-Mito was significantly dependent on the presence of ROS after ischemia-reperfusion

**FIGURE 5:**
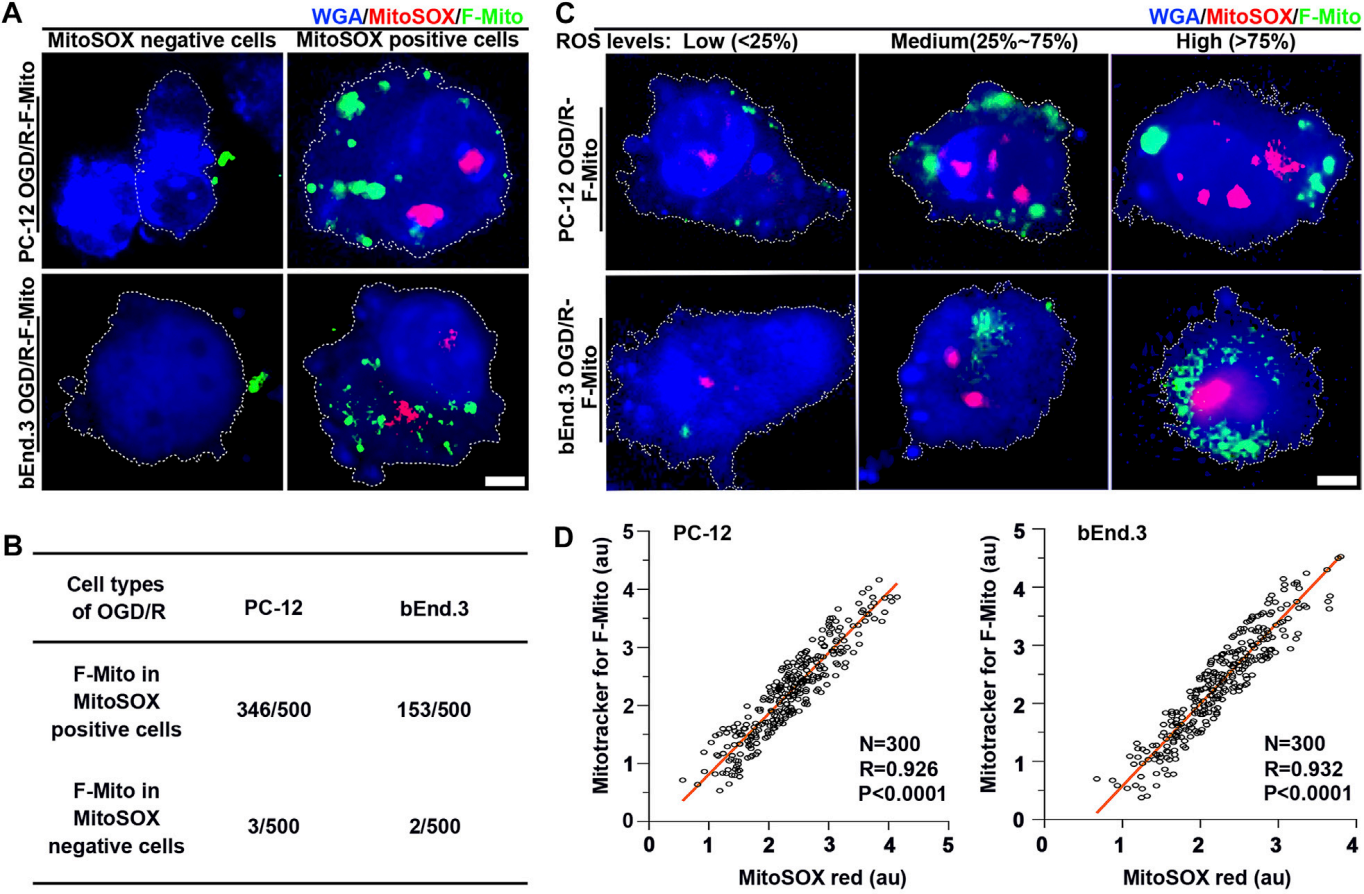
The F-Mito internalization correlated with ROS generation at the single cell level. Specifically, the results showed that cells with lower ROS levels exhibited decreased uptake of F-Mito. **(A)** Fluorescent images of PC-12 and bEnd.3 cells with and without F-Mito, respectively, after 9 h of OGD/R, and when cells were MitoSOX red positive or negative. The F-Mito were labeled with Mitotracker green, and cells were stained with WGA blue for visualization. Scale bars: 5 μm. **(B)** The uptake proportion of F-Mito in MitoSOX positive and negative cells at 9 h post OGD/R. 500 cells in each group were randomly selected in a uniform manner. **(C)** Representative fluorescent images of F-Mito uptake under different ROS levels at 6 h, as related to the boxplots presented in Supplementary Figures S16A, B, respectively. ROS were stained with MitoSOX red, F-Mito were labeled with Mitotracker green, and cells were stained with WGA blue for visualization. Scale bars: 5 μm. **(D)** A linear correlation between the internalized F-Mito level and ROS levels in OGD/R-stressed PC-12 and bEnd.3 cells at 6 h. Each data point represented a single cell, and the entire plot was based on measurements of 300 cells expressing MitoSOX selected in a uniform random manner. Pearson’s correlation coefficient (R) was reported, and mean gray value was presented as logs base e.

Given that dysfunctional mitochondria are the primary source of excessive ROS production, and functional mitochondria generate lower ROS levels, we probed whether ROS levels could trigger the uptake of exogenous healthy mitochondria at the single-cell level. We initially analyzed the uptake proportion of F-Mito in MitoSOX-positive or -negative PC-12/bEnd.3 cells, respectively, at 9 h post OGD/R. DsRed-labeled F-Mito were mostly found in MitoSOX-positive cells (Figures 5A, B and Supplementary Figure S15). Subsequently, we investigated whether F-Mito entry would vary with changing ROS levels. At 6 h, when ROS levels began to escalate in MitoSOX-positive PC-12 and bEnd.3 cells, the changes in ROS generation across 300 random cell samples were within an order of magnitude, and the linear proportionality between internalized F-Mito levels and ROS levels was maintained for the order of magnitude (Supplementary Figure S16 and Figures 5C, D). These findings suggest that higher ROS levels reinforce the ability of cells to internalize F-Mito. Thus, free healthy mitochondria are internalized by stressed cells only when ROS production increases, signifying a permissive event.

**FIGURE 6:**
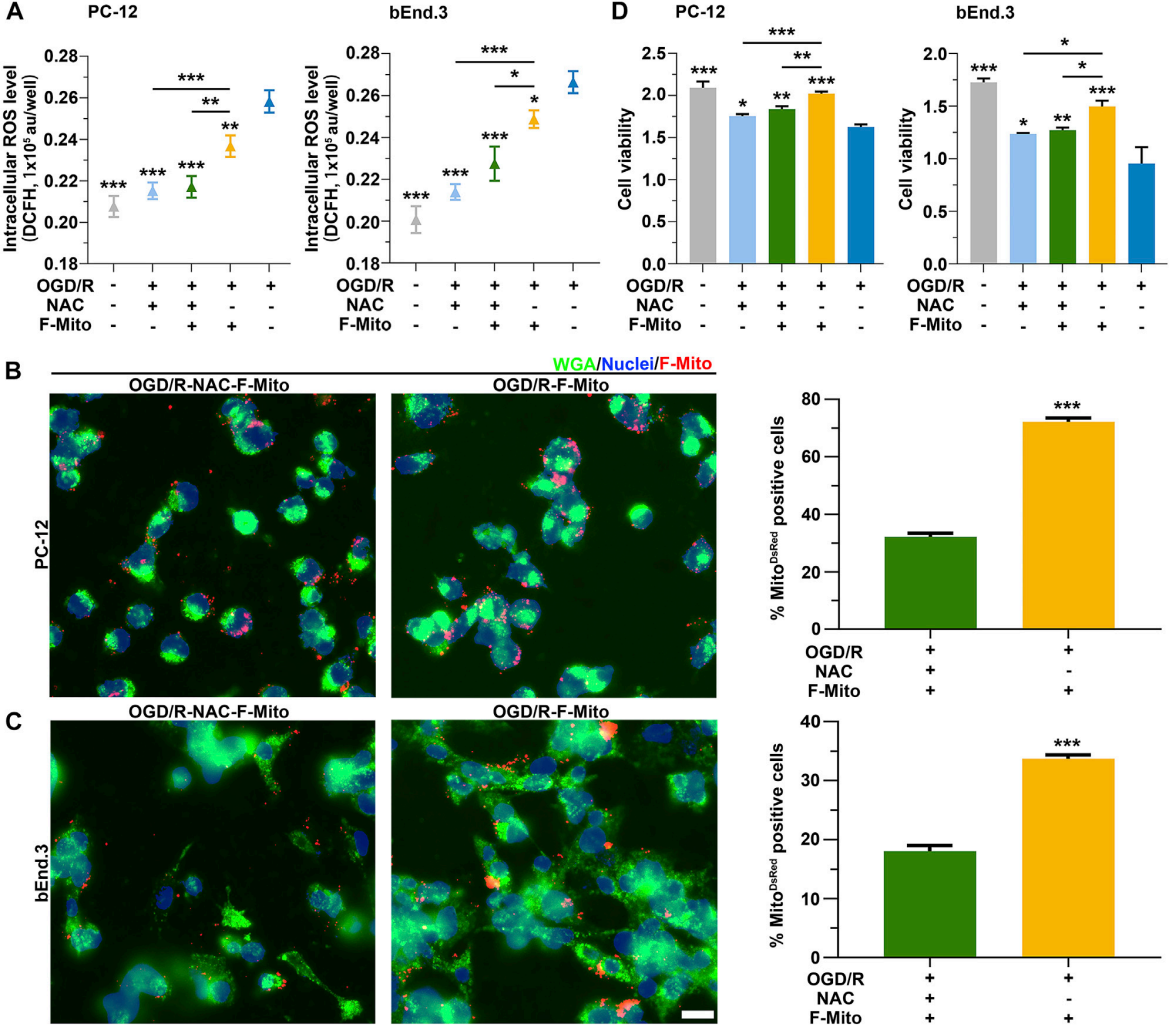
ROS was required to the uptake of F-Mito after ischemia reperfusion. **(A)** The results of intercellular ROS levels in PC-12 and bEnd.3 cells, respectively, after 9 h under different treatments. The cells, which were previously cultured under OGD/R, were treated with either NAC alone (OGD/R-NAC), NAC plus F-Mito (OGD/R-NAC-F-Mito), or F-Mito alone (OGD/R-F-Mito). Statistical analysis was conducted using One-way ANOVA with Bonferroni correction, and significance was considered when samples were compared to the OGD/R group or in comparisons between two different samples under a given line (*p < 0.05, **p < 0.01, ***p < 0.001). The data were presented as means ± s.d., n = 3 independent experiments. **(B,C)** Fluorescent images were captured to illustrate the entry of F-Mito into PC-12 and bEnd.3 cells, respectively, after 9 h in the OGD/R-NAC-F-Mito or OGD/R-F-Mito group. The statistics of the comparison of the F-Mito positive cell proportion in PC-12 and bEnd.3 cells were presented on the right side of the panels. All statistical analyses were performed using Student’s t-test with a significance level of *p < 0.05, **p < 0.01, and ***p < 0.001. The data were presented as means ± s.d. and were obtained from more than 100 cells across three independent experiments. Scale bars: 20 μm. **(D)** The results of cell viability in PC-12 and bEnd.3 cells, respectively, after 9 h under different treatments. *p < 0.05, **p < 0.01, ***p < 0.001, One-way ANOVA with Bonferroni correction. Means ± s.d., n = 3 independent experiments.

To validate the requirement of ROS for the internalization of F-Mito, we administered a ROS inhibitor, acetylcysteine (NAC), to cells undergoing OGD/R. As anticipated, ROS levels in PC-12 or bEnd.3 cells challenged with OGD/R were diminished upon treatment with NAC alone or in combination with F-Mito at 6 h (Supplementary Figure S17A) and at 9 h (Figure 6A). This decrease in ROS levels led to a reduction in the uptake proportion of F-Mito in NAC-treated cells relative to cells treated with F-Mito alone after OGD/R (Supplementary Figures S17B, C and Figures 6B, C), indicating the indispensability of ROS generation for the entry of free functional mitochondria. While the viability of stressed cells was restored upon NAC or F-Mito treatment, F-Mito alone-treated cells exhibited more remarkable survival than NAC alone-treated cells (Supplementary Figure S17D and Figure 6D) in PC-12 and bEnd.3. Similarly, ROS levels in neurons after OGD/R were decreased upon treatment with NAC alone or in combination with F-Mito at 6 h (Supplementary Figure S18) and led to a reduction in the uptake of F-Mito in NAC-treated neurons relative to that treated with F-Mito alone after OGD/R. The flow cytometry analysis also showed the reduction of F-Mito internalization when PC-12, bEnd.3 and neurons were treated with NAC at 9 h after OGD/R (Supplementary Figure S19).

The above results suggest ROS production is necessary to uptake F-Mito in stressed cells after ischemia-reperfusion. ROS levels correlate with the proportion of cells that internalize F-Mito, and the inhibition of ROS generation leads to decreased F-Mito uptake. Additionally, we observed that the F-Mito failed to enter cells with lower ROS levels after ischemia-reperfusion. These results highlight the potential therapeutic value of utilizing exogenous functional mitochondria to treat ischemia disease.

## 4 Discussion

Mitochondria, known as the “energy factory,” is an essential organelle in the cytoplasm that plays a crucial role in generating adenosine triphosphate (ATP) and maintaining cellular homeostasis. Recently, researchers have discovered that mesenchymal stem cells (MSCs) can transfer functional mitochondria to nearby damaged cells, including MSCs (Li et al., 2017), cardiomyocytes (Mori et al., 2023), and neural cells (Li et al., 2019). These mitochondrial transfers improve the performance of recipient cells undergoing stress, such as ischemia and reperfusion, and regulate pathological processes.

However, the mechanism behind initiating and driving exogenous mitochondrial transfer remains a question that needs to be answered. ROS are by-products of the redox process that occurs in mitochondria every minute. ROS levels can increase dramatically in response to environmental stress, leading to oxidative stress and damaging cellular proteins, lipids, and DNA (Valko et al., 2007). ROS production also contributes significantly to ischemic injury in the brain (Abramov et al., 2007), resulting in necrosis and apoptotic cell death, eventually leading to brain injury. In addition, accumulated ROS in focal ischemia damages the blood-brain barrier (Xie et al., 2020) and further induces secondary brain tissue injury via the inflammatory response (Chen & Li, 2020).

ROS has been reported to be essential in removing endogenous dysfunctional mitochondria via migrasome or mitophagy (Baldelli et al., 2014; Jiao et al., 2021). This study first found that the ROS produced in stressed cells seems to be the early alarm event that further initiates exogenous functional mitochondrial transfer and uptake. Consequently, the functional mitochondria transfer almost occurred in the cells with elevated ROS, and the higher the ROS levels, the stronger the ability of cells to internalize the exogenous functional mitochondria. Specifically, mitochondrial uptake was recipient cytoplasmic ROS level-dependent in different cell types after ischemia-reperfusion, and their different uptake abilities were due to their additional ROS production. Moreover, ROS was shown to be a significant determinant of acquiring free functional mitochondria, indicating that ROS could be a key signal for mitochondrial-targeted therapy. Furthermore, we also found that exogenous mitochondria could improve stressed cell survival by downregulating ROS. This new mechanism confirmed that ROS also played a pivotal role in acquiring free functional mitochondria in stressed cells and simultaneously recovering redox balance.

In addition to the exciting founding elaborated above, this study also explored a new engineering method for functional mitochondria transfer. Conventional mitochondrial transfer occurs intercellularly, relying on the formation of directly intercellular tunneling nanotubes (TNTs), gap junction channels (GJCs), and indirectly extracellular vesicles (EVs) (Liu et al., 2022). However, these avenues are low efficient, requiring healthy cells’ delivery and subcellular structure (e.g., TNTs, GJCs, and EVs) formation. In contrast, the study adopted a new strategy that isolates functional mitochondria from healthy UCMSCs *in vitro*, which are stable and easily upscale producing. Moreover, this novel method does not need to concern the donor cells’ fate and delivery because MSCs administration *in vivo* may also risk being kidnapped by the tumor cells and supporting the tumor cells’ development (Lin W. et al, 2019). Hence, this method is a novel strategy for cell-free stem-based therapy, which is more efficient and much safer than conventional direct MSC-based stem cell therapy.

Although mitochondrial transplantation has a potential for clinical application, it still has several disadvantages: for instance, 1. mitochondrial DNA has less reliable repair mechanisms than chromosomal DNA, therefore mitochondrial DNA mutation accumulation could be a problem in mitochondrial transplantation therapy since mitochondrial DNA mutations might produce neoantigens capable of eliciting immune recognition and rejection (Deuse et al., 2019). 2. whether exogenous mitochondrial transplantation can cause exogenous DNA contamination has not been carefully examined, which could raise ethical problems. 3. increased circulating mitochondria in organ donors may promote allograft rejection (Lin L. et al, 2019). Therefore, the long-term safety of mitochondria therapy should be further studied. And the effect of mitochondria extracted from different cells should be further analyzed.

It was reported that the membrane internalization-related protein clathrin (Levoux et al., 2021) and integrin-mediated src signaling (Hayakawa et al., 2016) play important roles in mitochondrial internalization. We used qRT-PCR to quantify both clathrin and src mRNA levels in OGD/R cells and OGD/R + NAC treated cells, and the results (Supplementary Figures S20A, B) showed that the mRNA levels of both clathrin and src were significantly upregulated in OGD/R condition; whereas the ROS inhibitor NAC treatment significantly decreased the upregulated mRNA levels of the both genes. On the other hand, CD38 has been reported as a key regulator of mitochondrial internalization (Hayakawa et al., 2016; Wang Y. et al, 2020; Ni et al., 2022). Our qRT-PCR results also (Supplementary Figure S20C) showed that CD38 was significantly upregulated in OGD/R condition; whereas NAC treatment significantly decreased the upregulated mRNA levels of CD38 in mouse neurons, astrocytes, and endothelial cells. These results suggest that the genes that facilitate cell endocytosis may be involved in the cellular process of mitochondrial internalization regulated by ROS levels.

In conclusion, this study provides evidence that ROS levels, as an early response for mediating functional mitochondria internalization to restore cell viability after ischemia stroke, shedding light on the precise regulation of mitochondrial transplantation and developing a powerful method for therapeutic purposes in ischemia stroke. In addition, the techniques presented in this study suggest reliable functional mitochondrial transplantation could be an innovative and effective strategy to treat ischemic stroke, which could potentially help millions of people worldwide. However, future studies must further investigate the precise mechanisms and specific responsive molecules in downstream exogenous mitochondrial transfer and explore potential applications of mitochondria transfer to treat ischemia stroke.

## Data Availability

The raw data supporting the conclusion of this article will be made available by the authors, without undue reservation.
