# Supplementary material for: ROS-responsive exogenous functional mitochondria can rescue neural cells post-ischemic stroke
Source: Front Cell Dev Biol. 2023 Jul 3;11:1207748. doi: 10.3389/fcell.2023.1207748 (PMC10350566; doi:10.3389/fcell.2023.1207748)
Supplement: Supplementary file 1 [file DataSheet1.pdf]

Supplementary Material

ROS-responsive exogenous functional mitochondria can rescue neural cells post-ischemic stroke

Yanjiao Li[1,2†], Ya-chao Wang[3†] , Weiqi Yang[4†]， Zhen Wu[5], Daiping Ma[5], Jianxiu Sun[5]， Huixian Tao[5], Qinlian Ye[3]， Jingnan Liu[6]， Zhaoxia Ma[2], Lihua Qiu[2], Weiping Li[7*] ,Liyan Li[1*], Min Hu[2,5,8*]

[1]Institute of Neuroscience, Kunming Medical University, Kunming, China.
[2]Yunnan Key Laboratory for Basic Research on Bone and Joint Diseases & Yunnan Stem Cell Translational Research Center, Kunming University, Kunming, China.
[3]Department of Neurosurgery, The Institute Translational Medicine, The First Affiliated Hospital of Shenzhen University, Shenzhen Second People's Hospital, Shenzhen, China.
[4]Department of Burn plastic surgery，Shenzhen Second People's Hospital，Shenzhen， China.
[5]Yunnan Jici Institute for Regenerative Medicine Co., Ltd.
[6]Department of pathophysiology, Basic medical and public health school, Jinan University, Guangzhou, China.
[7]Department of Neurosurgery, Shenzhen Second People’s Hospital/The First Affiliated Hospital of Shenzhen University Health Science Center, Shenzhen, China.
[8]Shenzhen Zhendejici Pharmaceutical Research and Development Co., Ltd.

[†]These authors contributed equally to this work
*Correspondence :
✉e-mail: wpli@szu.edu.cn; kmliyanl@163.com; huminynkm@163.com.

1 Supplementary Figures

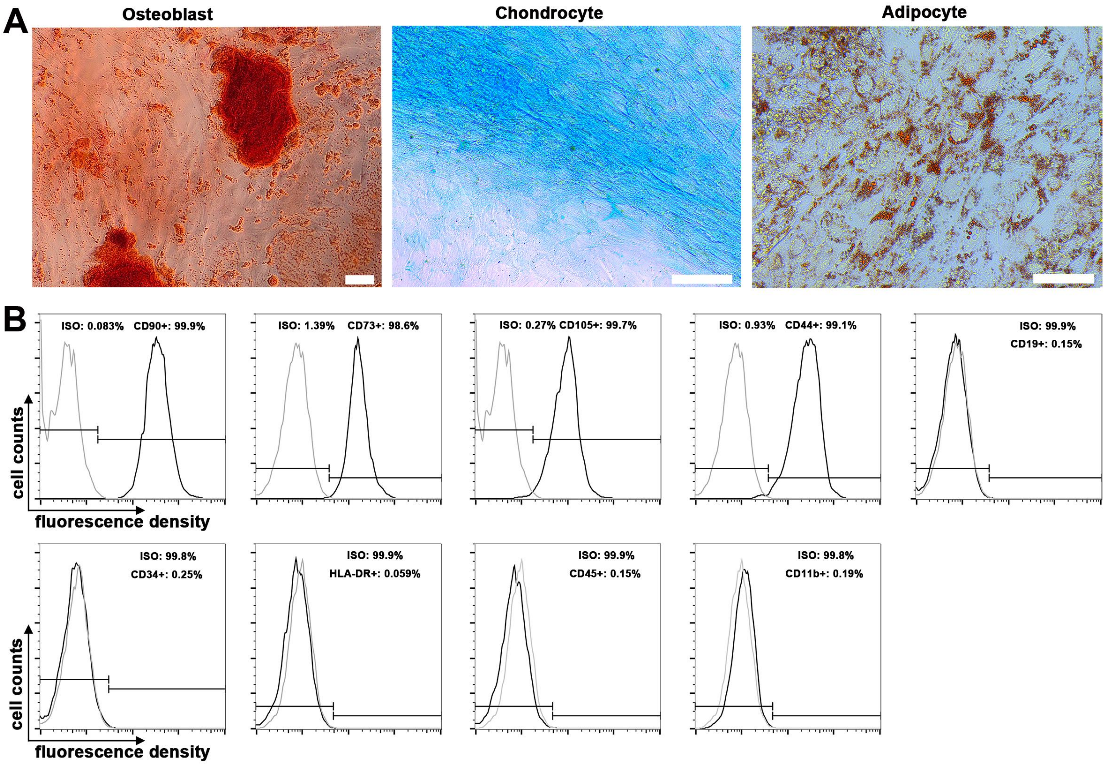

Supplementary Fig 1. Characterization of UCMSCs from which F-Mito were isolated

(A). Osteogenic, chondrogenic and adipogenic differentiation capabilities of human UCMSCs. Scale bars, 100 μm.

(B). Identification of positive and negative cell surface markers in human UCMSCs by FACS. More than 95% of the hUCMSCs were positive for CD90, CD105, CD44, CD73, and less than 2% of them were positive for CD45, CD19, CD34, CD11b, and HLA-DR.

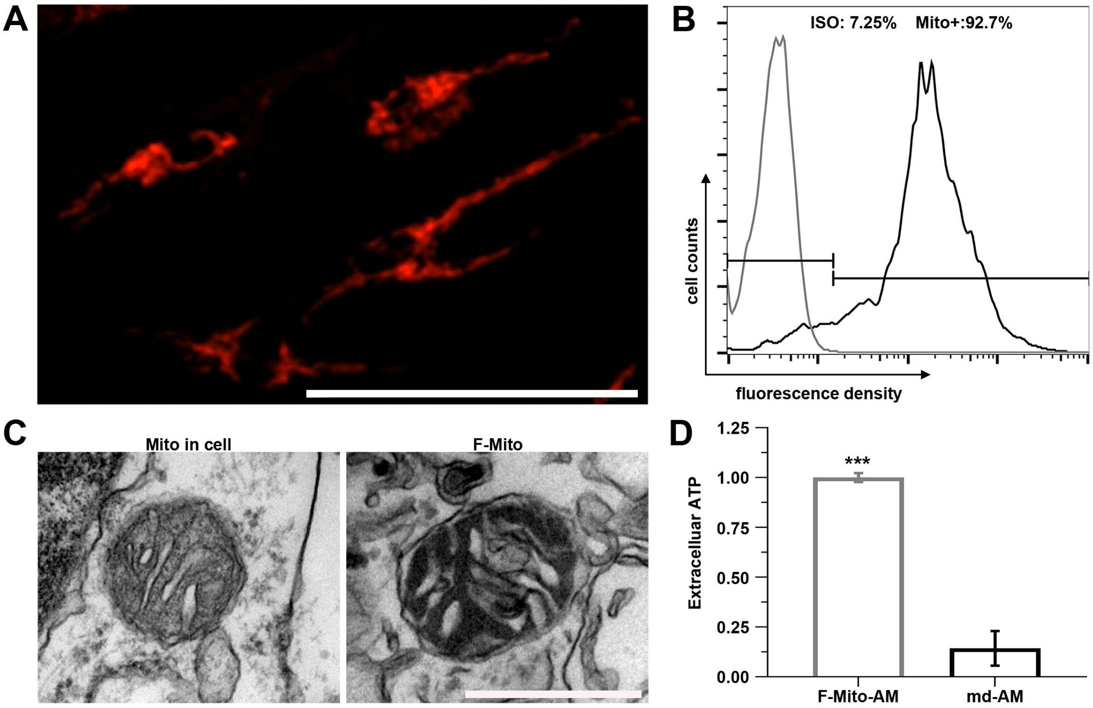

Supplementary Fig 2. Characterization of F-Mito extracted from UCMSCs

(A). Labeling mitochondria in UCMSCs with RFP (DsRed) by lentivirus. Scale bars, 100 μm.

(B). Efficiency of labeling in mitochondria was ≥ 90% by FACS.

(C). TEM images showed DsRed-labeled mitochondria had dense cristate and intact membranes, indicative of healthy mitochondria. Scale bars, 500 nm.

(D). The F-Mito showed robust ATP activity in vitro. **p* < 0.05, ***p* < 0.01, ****p* < 0.001, Student t-test. Means ± s.d., n= 3 independent experiments.

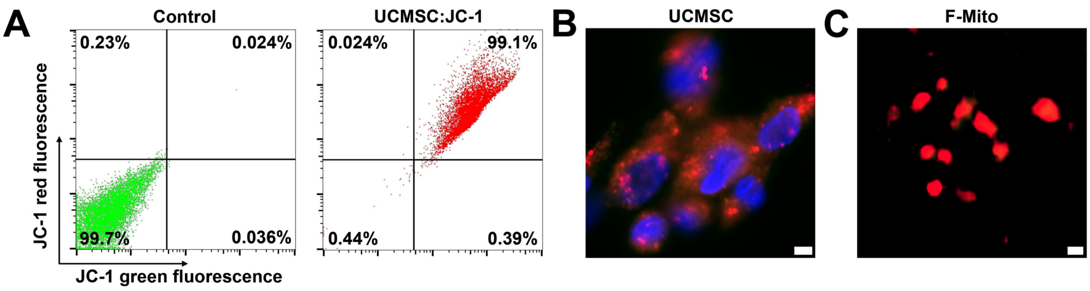

Supplementary Fig 3. Mitochondria membrane potential in UCMSCs and F-Mito

(A). Mitochondria membrane potential in UCMSCs were analyzed using JC-1 dye by FACs. Control without JC-1 staining.

(B). Mitochondrial membrane potential assessed in UCMSCs by a fluorescent microscopy. Red: aggregated JC1, Green: monomer JC1. Scale: 5 μm.

(C). Mitochondrial membrane potential assessed in F-Mito by a fluorescent microscopy. Red: aggregated JC1, Green: monomer JC1. Scale: 1 μm.

Supplementary Table 1 Mitochondrial Enzymatic activity and mtDNA Copy Number

**Samples**	**mtDNA copy number/cell**	**Citrate synthase activity (U/mgprot)**	**mitochondria complex Ⅳ activity (nmol/min/mg prot)**
UCMSCs	831 ± 142	1.58 ± 0.69	0.52 ± 0.09
F-Mito	/	1.79 ± 0.69	0.48 ± 0.18

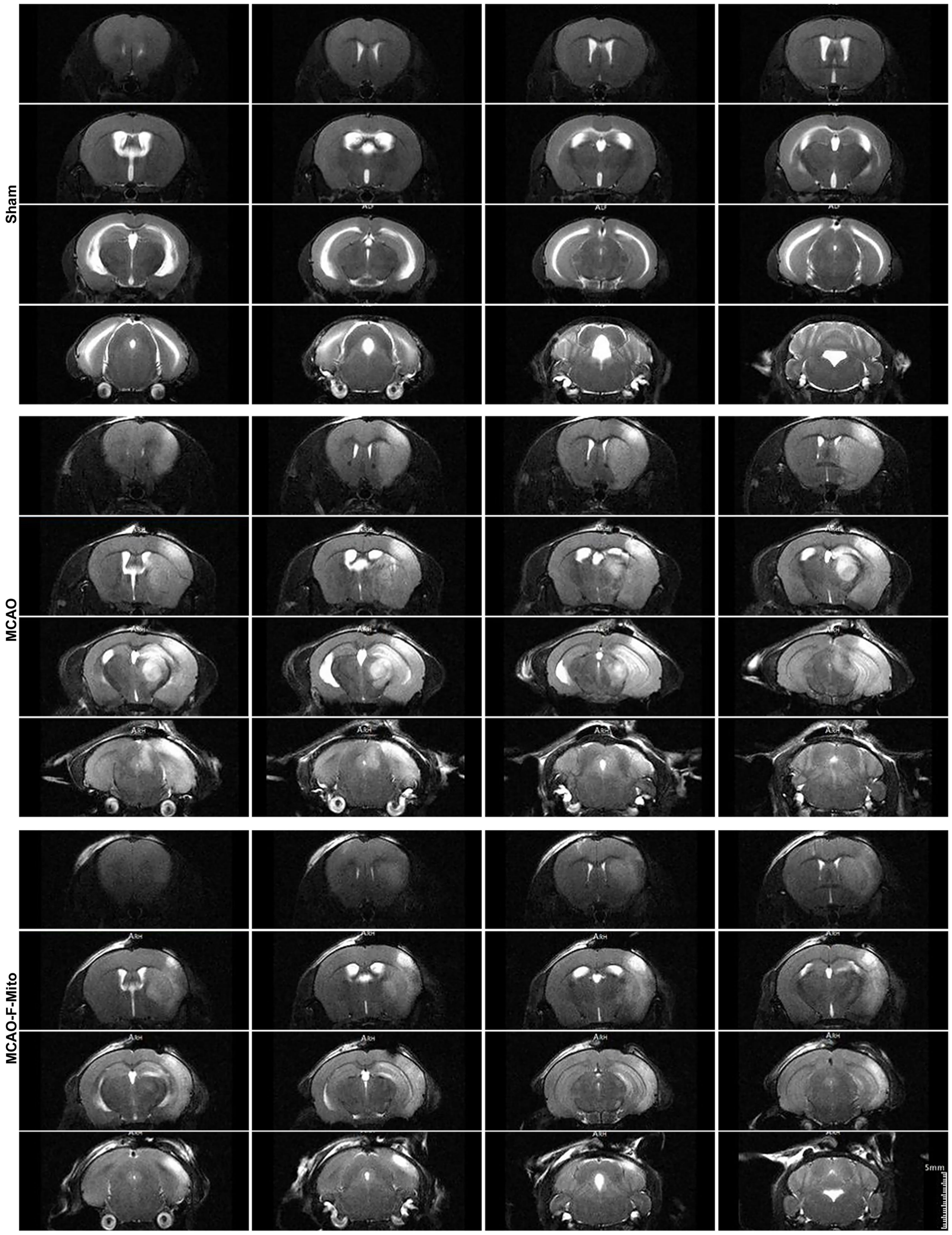

Supplementary Fig 4. MRI images of brain tissue on day 3 post-stroke.

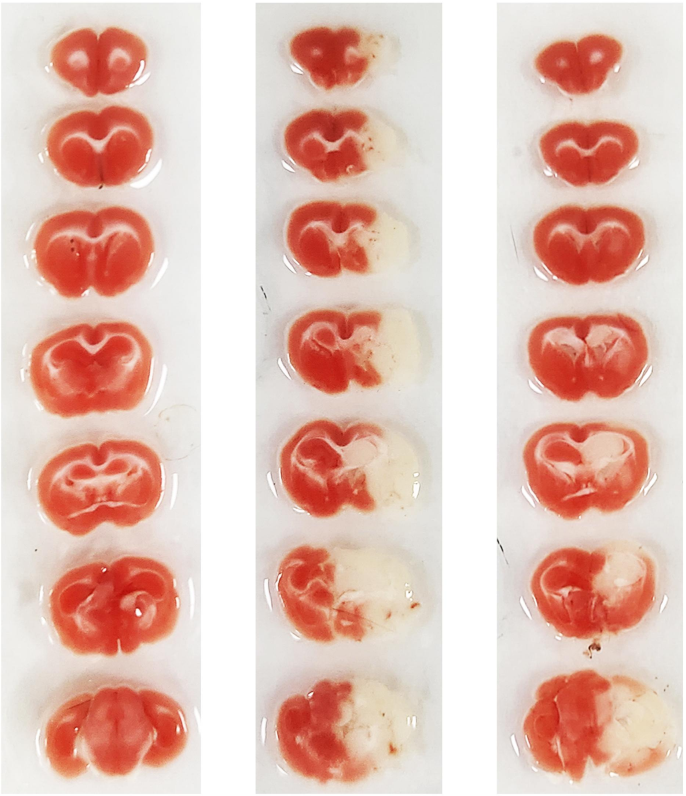

**MCAO**	-	+	+
**F-Mito**	-	-	+

Supplementary Fig 5. TTC staining of brain tissue on day 3 post-stroke.

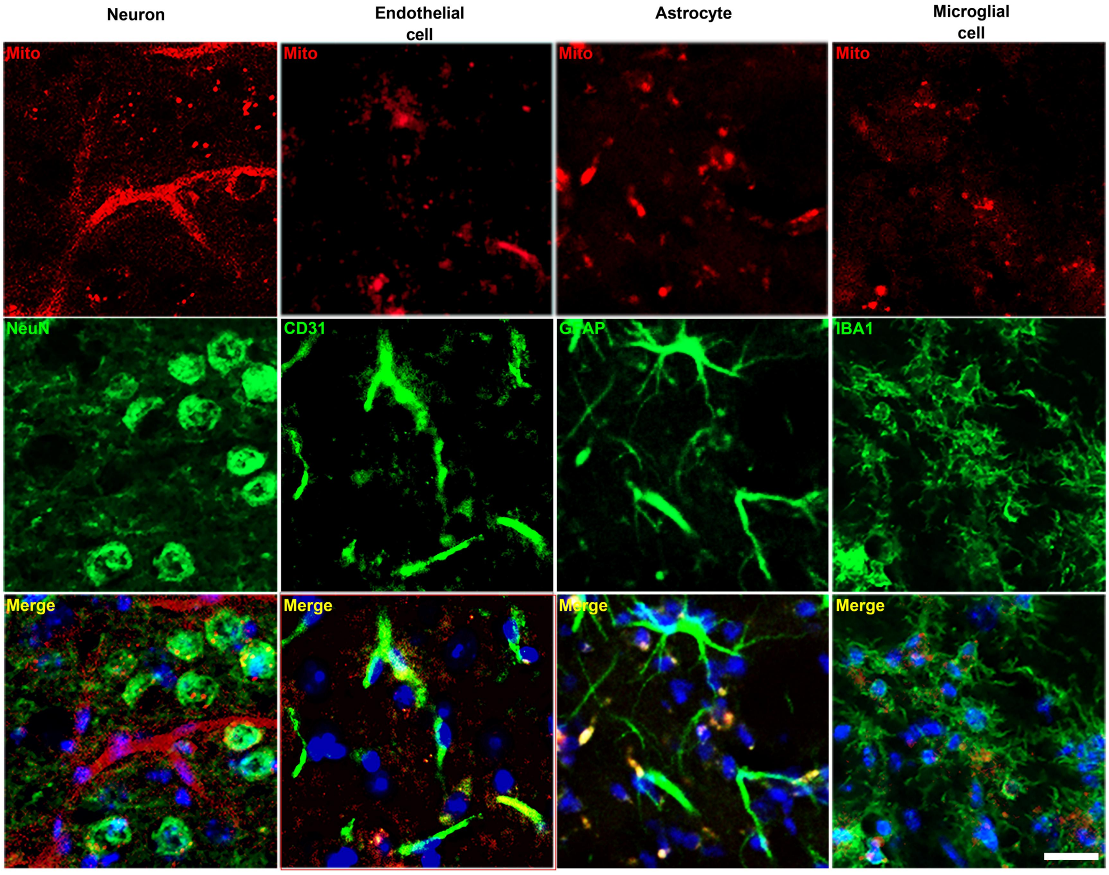

Supplementary Fig 6. The uptake of F-Mito by neurons, endothelial cells, astrocytes and microglial cells in brain 28 days after ischemia stroke.

The confocal images presented in this figure demonstrated the internalization of F-Mito into cells within the lesion of the injection area. Scale bar: 20 μm.

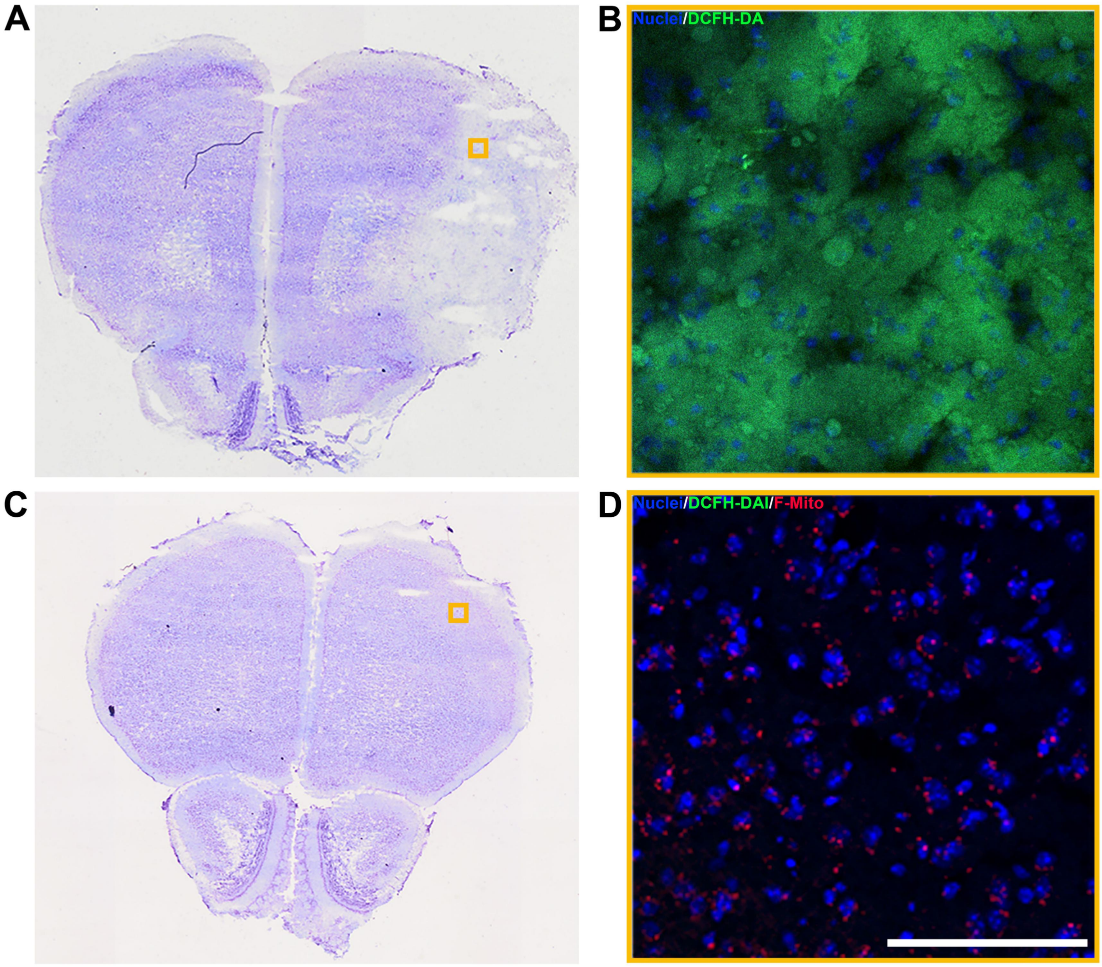

Supplementary Fig 7. The ROS levels in mice brain tissues

(A). Nissl staining showed the injured area in MCAO group 3 days post surgery.

(B). The ROS levels in the injured area in MCAO group, assessed by the ROS probe DCFH-DA (green).

(C). Nissl staining showed the injured area in MCAO-F-Mito group 3 days post surgery.

(D). The ROS levels in the injured area in MCAO-F-Mito group, assessed by the ROS probe DCFH-DA (green). Scale bar: 100 μm.

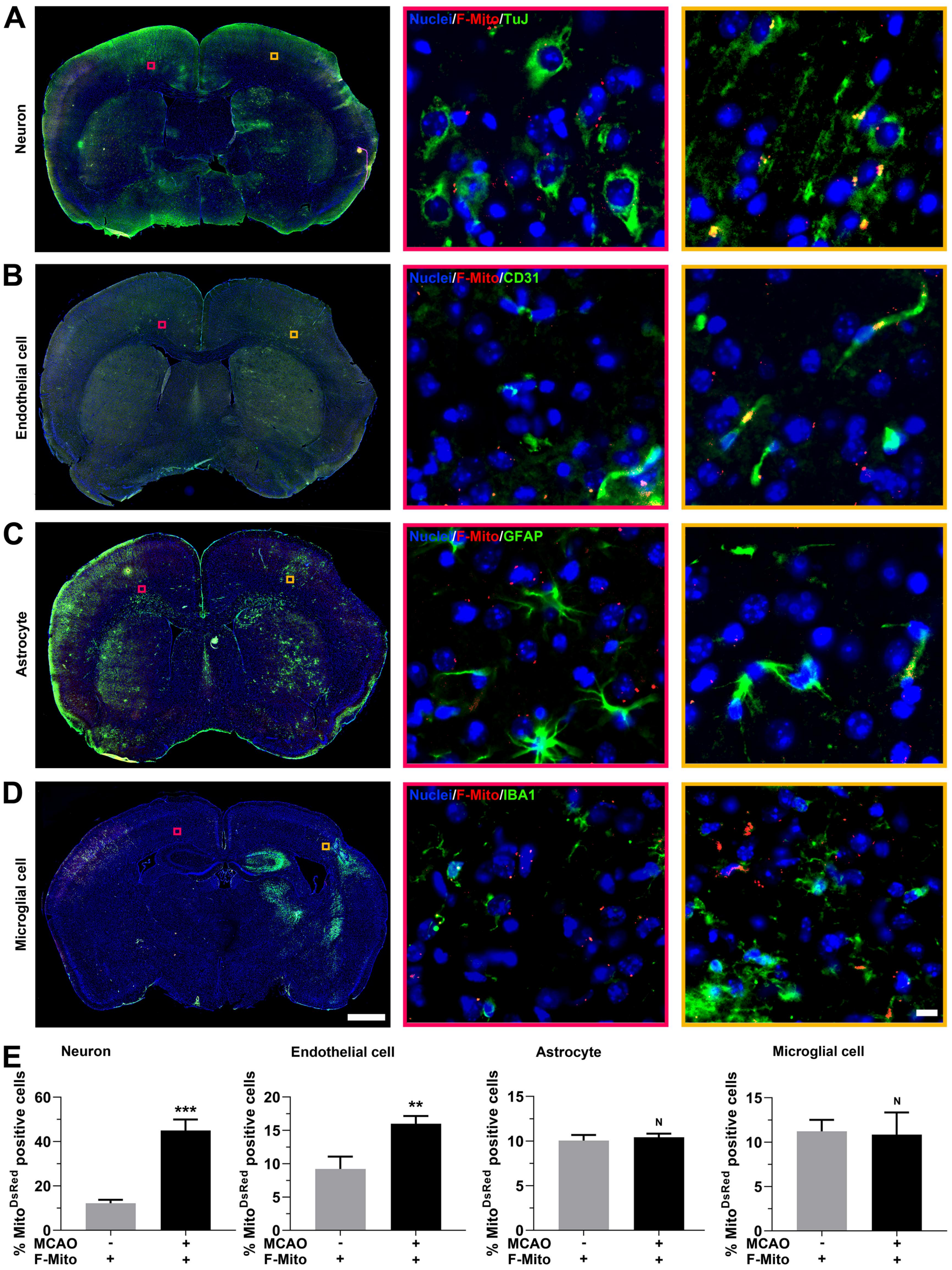

Supplementary Fig 8. The comparison of F-Mito internalization by neurons, endothelial cells, astrocytes and microglial cells between normal brain and infarcted brain after ischemia stroke.

(A). The Fluorescent images demonstrated the internalization of F-Mito into Neurons within normal brain area (red rectangle) and the lesion area (yellow rectangle) 28 days post MCAO. Scale bar for whole brain: 1000 μm. Scale bar for magnification images: 10 μm.

(B). The Fluorescent images demonstrated the internalization of F-Mito into endothelial cells

within normal area (red rectangle) and the lesion area (yellow rectangle).

(C). The Fluorescent images demonstrated the internalization of F-Mito into astrocytes within normal area (red rectangle) and the lesion area (yellow rectangle).

(D). The Fluorescent images demonstrated the internalization of F-Mito into microglial cells within normal area (red rectangle) and the lesion area (yellow rectangle).

(E). The proportion of cells with F-Mito in brain was represented, indicating statistically significant differences in comparisons made between the normal and injured brain tissues. The level of significance was denoted by *$p < 0.05$, **$p < 0.01$, and ***$p < 0.001$, as determined by Student t-test. The data were presented as means ± standard deviation (s.d.), and the study was conducted on three animals in each group.

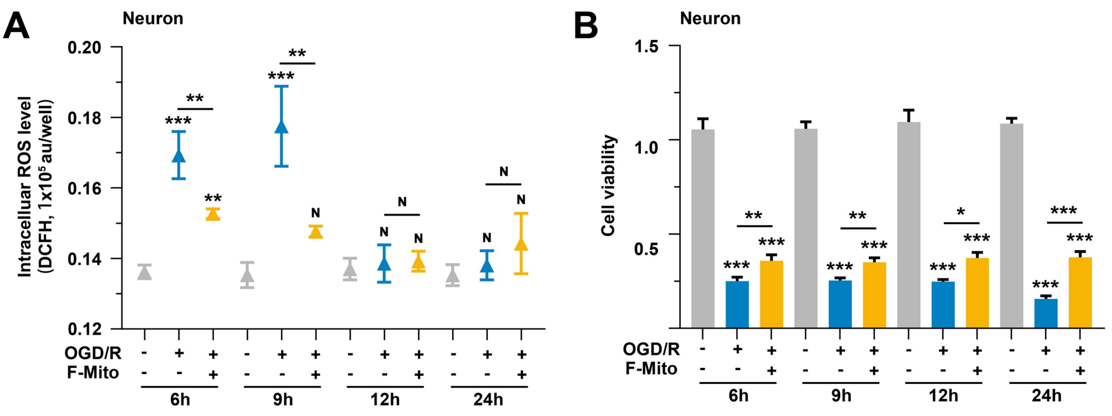

Supplementary Fig 9. The treatment of F-Mito reduced intracellular ROS and restored cell viability after OGD/R in neurons.

The study was performed on primary neurons, and the results were reported for a period of 24 hours after OGD/R. The treatment with F-Mito led to a restoration of cell viability and a reduction in intracellular ROS levels.

(A-B). The intracellular ROS levels and cell viability in mice primary neurons over time after OGD/R. Statistical analysis was conducted using One-way ANOVA with Bonferroni correction, and significance was considered when samples were compared to the control group at their respective time points (*$p < 0.05$, **$p < 0.01$, ***$p < 0.001$). The data were presented as means ± s.d., n = 3 independent experiments.

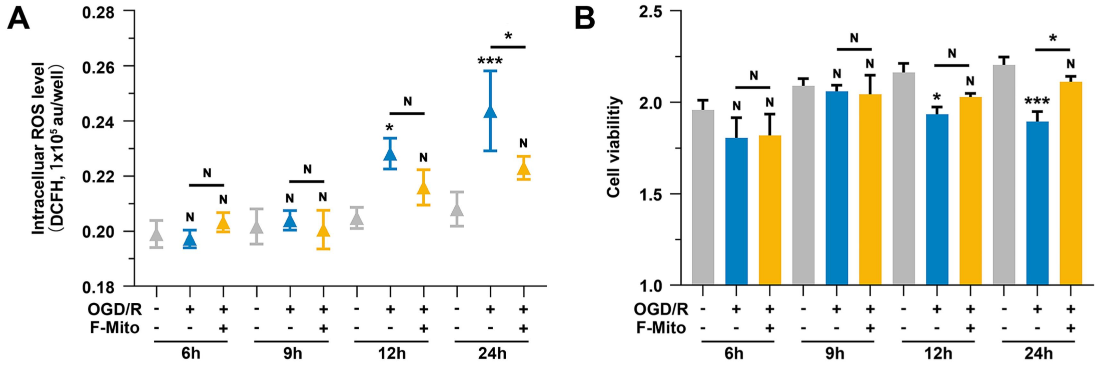

Supplementary Fig 10. The treatment of F-Mito reduced intracellular ROS and restored cell viability after OGD/R in astrocytes.

The study was performed on astrocytes, and the results were reported for a period of 24 hours after OGD/R. The treatment with F-Mito led to a restoration of cell viability and a reduction in intracellular ROS levels.

(A-B). The intracellular ROS levels and cell viability in astrocytes over time after OGD/R. Statistical analysis was conducted using One-way ANOVA with Bonferroni correction, and significance was considered when samples were compared to the control group at their respective time points (**p* < 0.05, ***p* < 0.01, ****p* < 0.001). The data were presented as means ± s.d., n = 3 independent experiments.

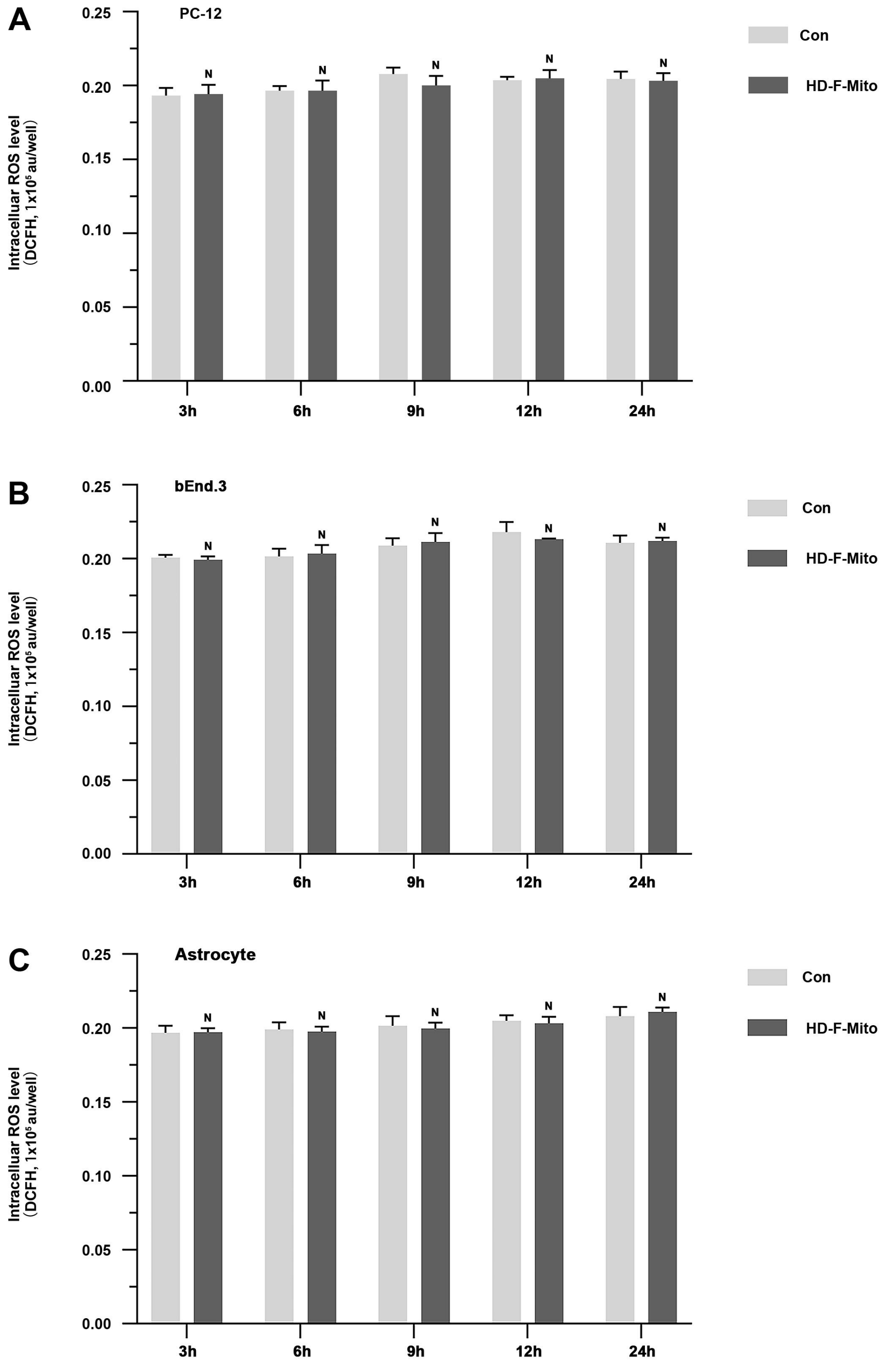

Supplementary Fig 11. ROS levels in normal PC-12, bEnd.3 and astrocytes after treated by F-Mito.

Significance was determined when samples were compared to the control group at their respective time point. *p < 0.05, **p < 0.01, ***p < 0.001. Student t test, Means ± s.d., n = 3

independent experiments. Con: cells were cultured under normal conditions, HD-Mito: cells were cultured under normal conditions and added F-Mito.

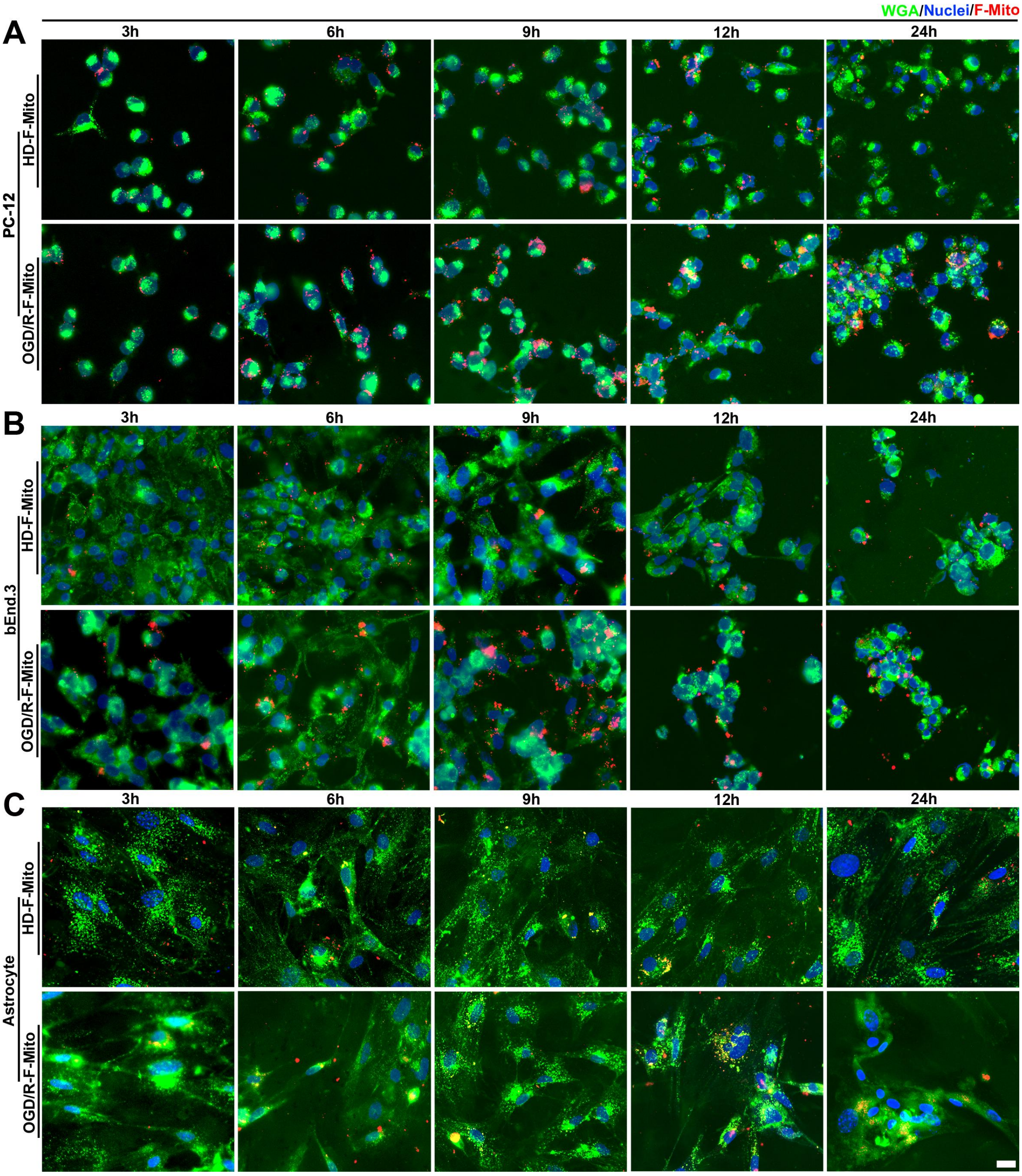

Supplementary Fig 12. Fluorescent images showed that the uptake of F-Mito by (A) PC-12, (B) bEnd.3, (C) astrocytes over time within 24 hours. Scale bars, 20 μm.

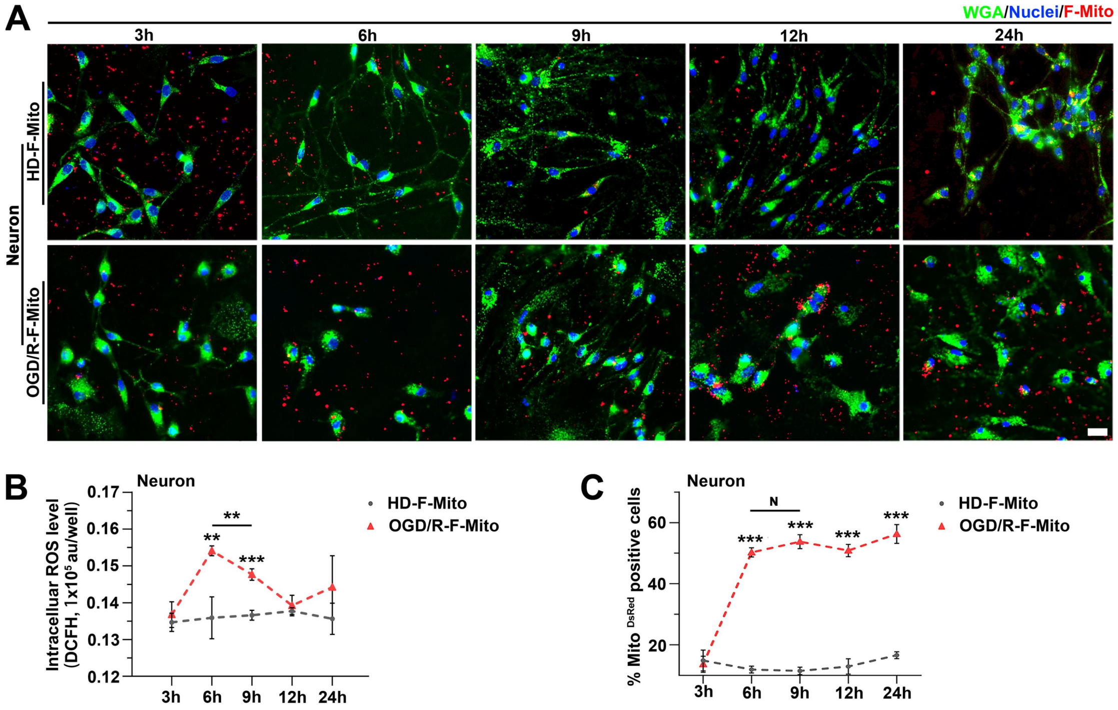

Supplementary Fig 13. The uptake of F-Mito by primary neurons.

(A) Fluorescent images showed the uptake of F-Mito by neurons. Scale bars, 20 μm.

(B) The intracellular ROS production within 24 hours in neurons after OGD/R.

(C) The proportion of neuron with F-Mito over time. Statistical analysis was conducted using Student t-test and significance was determined when sample in OGD/R-F-Mito group was compared to that in HD-F-Mito at different time point or in comparisons between two different samples under a given line (**p* < 0.05, ***p* < 0.01, ****p* < 0.001).The data were presented as means ± standard deviation (s.d.), n = 3 independent experiments in ROS detection, and n > 100 cells from three independent experiments in the analyses of the Mito^{DsRed} positive cell ratio .

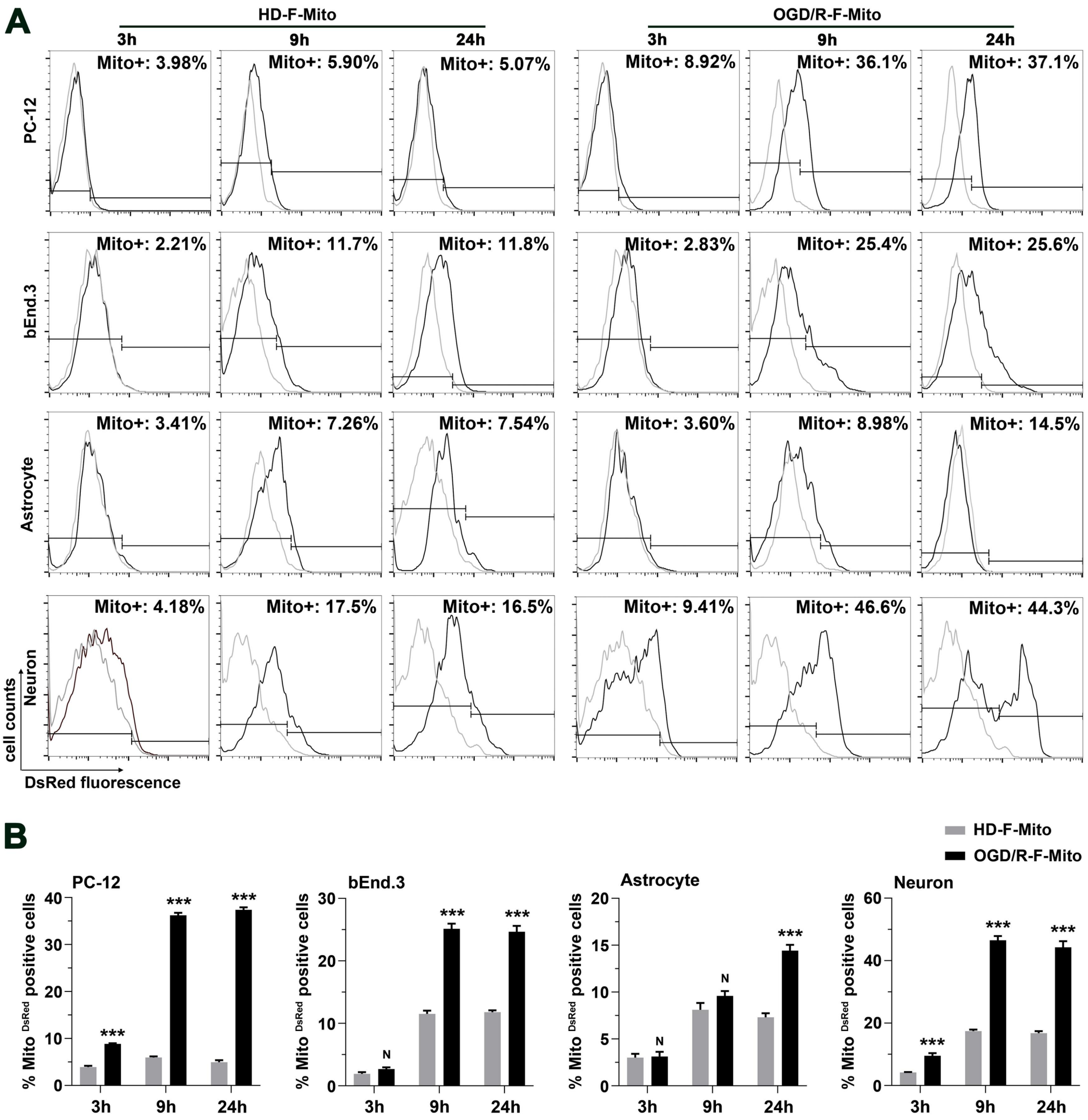

Supplementary Fig 14. Flow cytometry analysis showed the proportion of the cells with F-Mito.

(A). Efficiency of the PC-12, bEnd.3, astrocytes and neurons with F-Mito by FACS.

(B). The comparison of F-Mito positive cell percentage at 3 hours, 9 hours and 24 hours using Flow cytometry analysis. The statistical analyses were performed using one-way ANOVA with Bonferroni correction, and the significance levels were set at *$p < 0.05$, **$p < 0.01$, and ***$p < 0.001$. The data were presented as means ± s.d. and were obtained across three independent experiments.

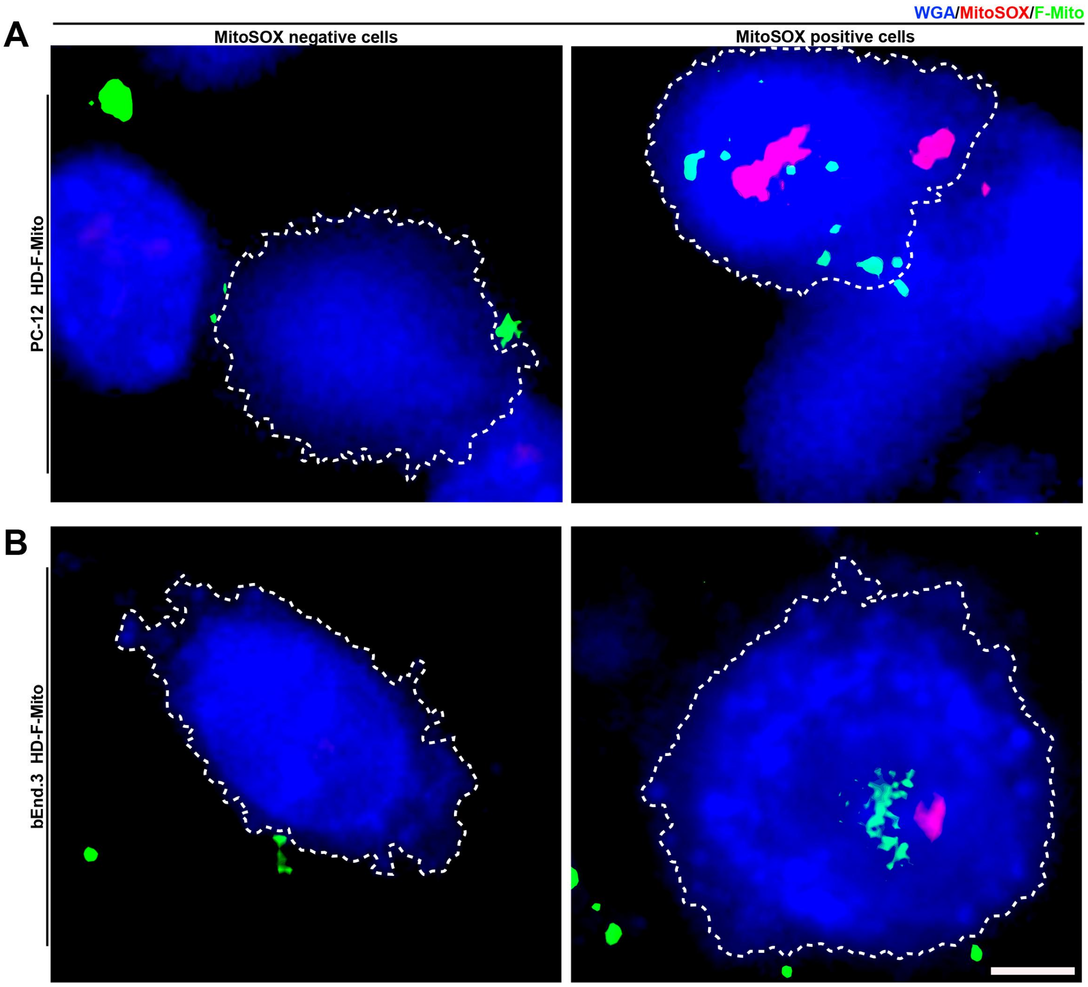

Supplementary Fig 15. The F-Mito internalization in the normal cells with or without excessive ROS.

(A). Fluorescent images showed PC-12 cultured under the normal condition with and without F-Mito, respectively, and when cells were MitoSOX red positive or negative.

(B). Fluorescent images showed bEnd.3 cultured under the normal condition with and without F-Mito, respectively, and when cells were MitoSOX red positive or negative. The F-Mito were labeled with Mitotracker green, and cells were stained with WGA blue for visualization. Scale bars，5 μm.

A

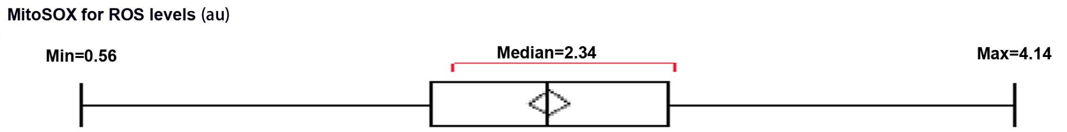

B

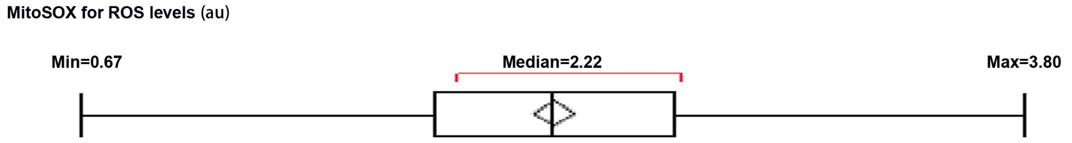

Supplementary Fig 16. Quartile of ROS levels shown by MitoSOX red fluorescence intensity in OGD/R stressed PC-12 and bEnd.3 cells.

(A). Quartile of ROS levels were shown in OGD/R stressed PC-12 cells at 6 hours. Minimum, maximum and median value was 0.56, 4.14 and 2.34 respectively.

(B). Quartile of ROS levels were shown in OGD/R stressed bEnd.3 cells at 6 hours. Minimum, maximum and median value was 0.67, 3.80 and 2.22 respectively. Mean gray value was presented as logs base e.

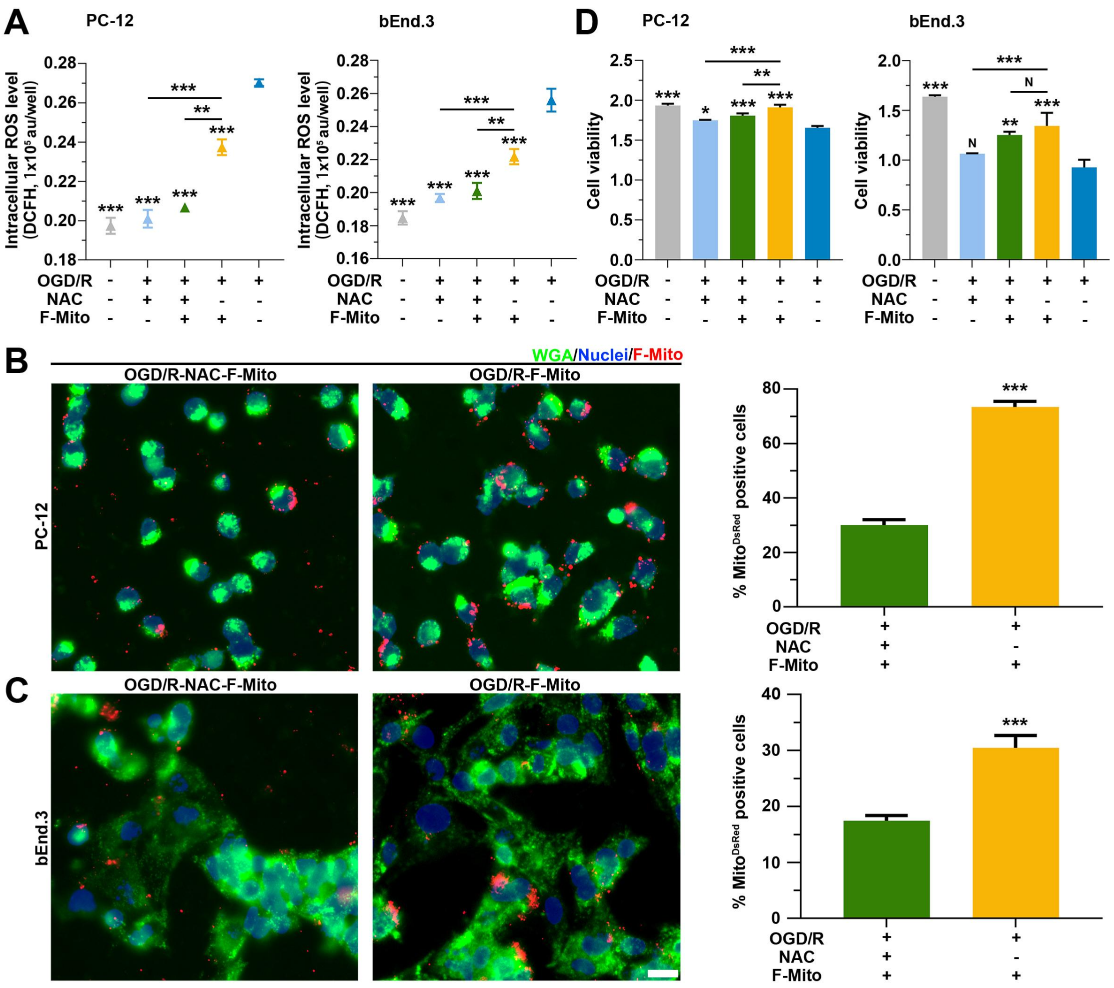

Supplementary Fig 17. ROS was required to the uptake of F-Mito after ischemia reperfusion.

(A). The results of intercellular ROS levels in PC-12 and bEnd.3 cells, respectively, after 6 hours under different treatments. The cells, which were previously cultured under OGD/R, were treated with either NAC alone (OGD/R-NAC), NAC plus F-Mito (OGD/R-NAC-F-Mito), or F-Mito alone (OGD/R-F-Mito). Statistical analysis was conducted using One-way ANOVA with Bonferroni correction, and significance was considered when samples were compared to the OGD/R group or in comparisons between two different samples under a given line (**p* < 0.05, ***p* < 0.01, ****p* < 0.001). The data were presented as means ± s.d., n = 3 independent experiments.

(B-C). Fluorescent images were captured to illustrate the entry of F-Mito into PC-12 and bEnd.3 cells, respectively, after 6 hours in the OGD/R-NAC-F-Mito or OGD/R-F-Mito group. The statistics of the comparison of the F-Mito positive cell proportion in PC-12 and bEnd.3 cells were presented on the right side of the panels. All statistical analyses were performed

using Student t-test with a significance level of *$p < 0.05$, **$p < 0.01$, and ***$p < 0.001$. The data were presented as means ± s.d. and were obtained from more than 100 cells across three independent experiments. Scale bars: 20 μm.

(D). The results of cell viability in PC-12 and bEnd.3 cells, respectively, after 6 hours under different treatments. *$p < 0.05$, **$p < 0.01$, ***$p < 0.001$, One-way ANOVA with Bonferroni correction. Means ± s.d., n = 3 independent experiments.

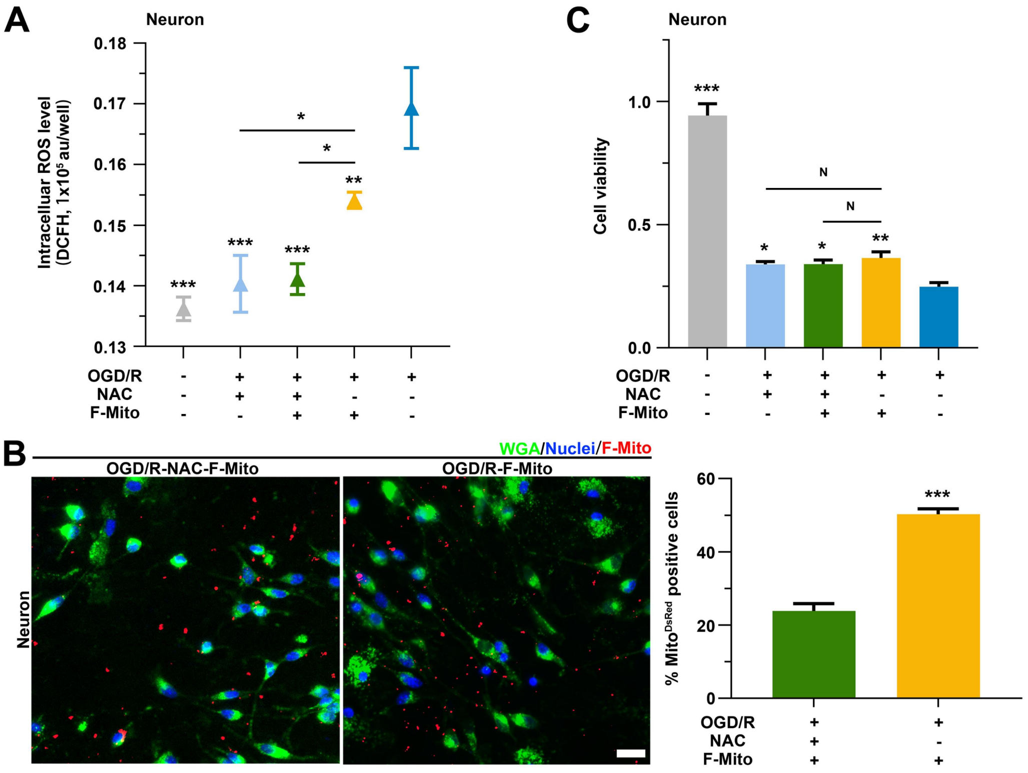

Supplementary Fig 18. ROS was required to the uptake of F-Mito by neurons after OGD/R.

(A) The results of intercellular ROS levels in neurons, after 6 hours under different treatments. The cells, which were previously cultured under OGD/R, were treated with either NAC alone (OGD/R-NAC), NAC plus F-Mito (OGD/R-NAC-F-Mito), or F-Mito alone (OGD/R-F-Mito). Statistical analysis was conducted using One-way ANOVA with Bonferroni correction, and significance was considered when samples were compared to the OGD/R group or in comparisons between two different samples under a given line (*$p < 0.05$, **$p < 0.01$, ***$p < 0.001$). The data were presented as means ± s.d., n = 3 independent experiments

(B) Fluorescent images were captured to illustrate the entry of F-Mito into neurons after 6 hours in the OGD/R-NAC-F-Mito or OGD/R-F-Mito group. Scale bars: 20 μm. Statistical

analyses were performed using Student t-test with a significance level of *p < 0.05, **p < 0.01, and ***p < 0.001. The data were presented as means ± s.d. and were obtained from more than 100 cells across three independent experiments.

(C) The results of cell viability in neurons after 6 hours under different treatments. *p < 0.05, **p < 0.01, ***p < 0.001, One-way ANOVA with Bonferroni correction. Means ± s.d., n = 3 independent experiments.

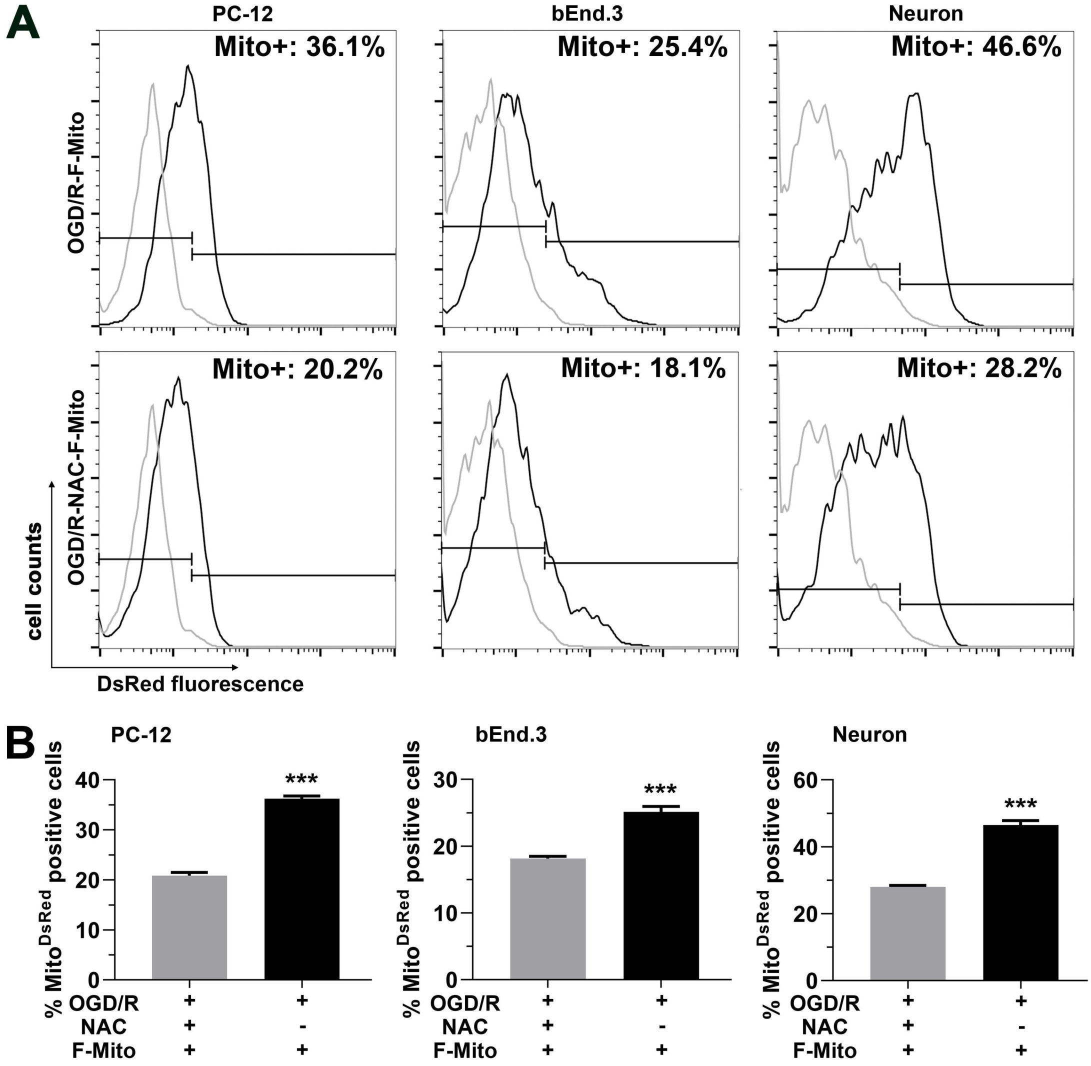

Supplementary Fig 19. Flow cytometry analysis showed the proportion of the F-Mito positive cells with or without NAC 9 hours after OGD/R.

(A). Efficiency of the PC-12, bEnd.3 and neurons with F-Mito by FACS in OGD/R-F-Mito group or in OGD/R-NAC-F-Mito group at 9 hours post OGD/R .

(B). The comparison of F-Mito positive cell percentage at 9 hours using Flow cytometry analysis. The statistical analyses were performed using Student t-test, and the significance

levels were set at *$p < 0.05$, **$p < 0.01$, and ***$p < 0.001$. The data were presented as means ± s.d. and were obtained across three independent experiments.

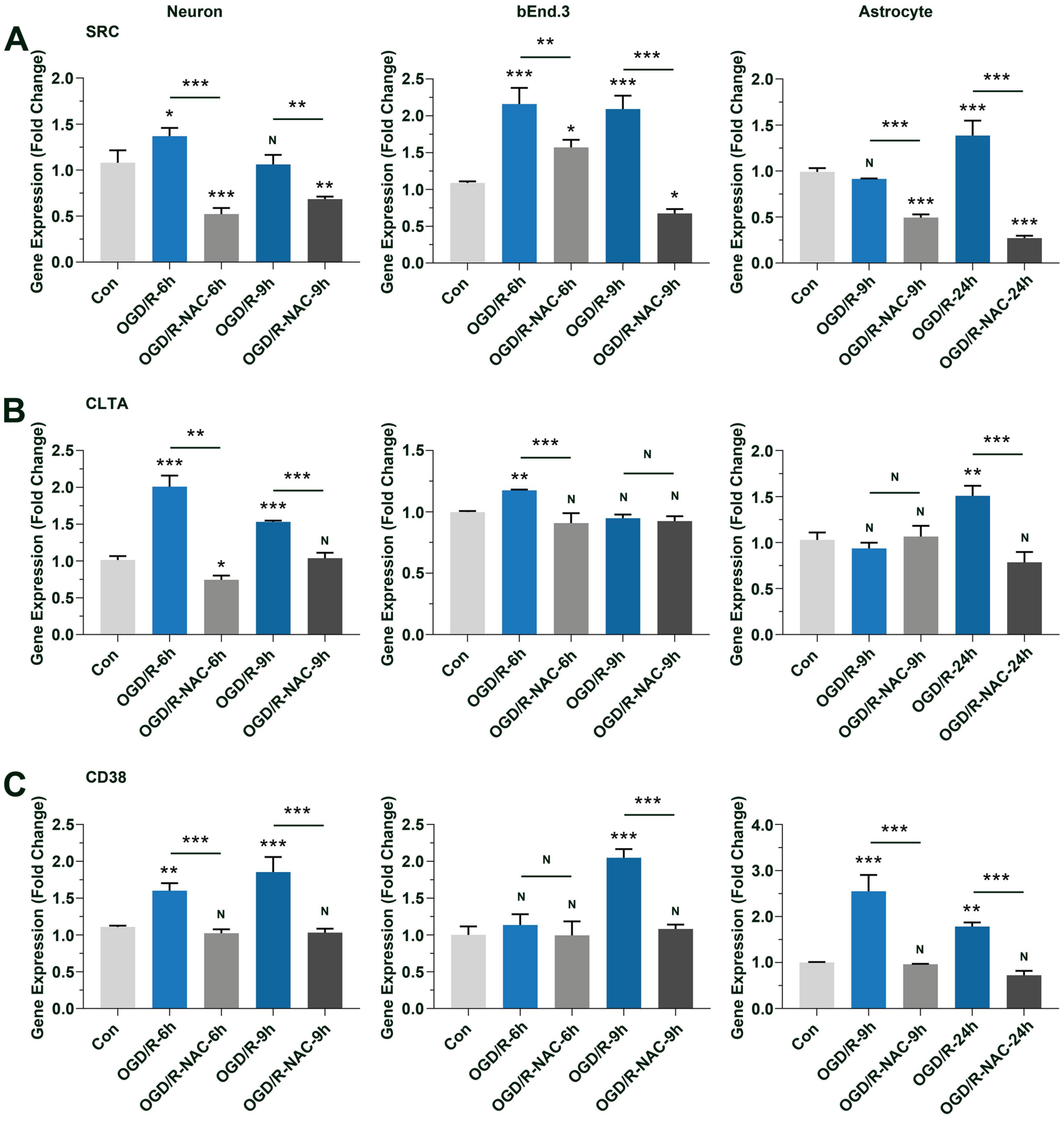

Supplementary Fig 20. qRT-PCR analysis of SRC, CLTA and CD38 expression in neurons, bEnd.3 and astrocytes.

(A-C). *SRC, CLTA* and *CD38* expression in neurons, bEnd.3 and astrocytes with or without NAC treatment overtime after OGD/R. The statistical analyses were performed using one-way ANOVA with Bonferroni correction, and the significance levels were considered when samples were compared to the control group or in comparison between two different samples under a given line(*$p < 0.05$, **$p < 0.01$, and ***$p < 0.001$). The data were presented as means ± s.d. and were obtained across three independent experiments.
